# Screening of world approved drugs against highly dynamical spike glycoprotein of SARS-CoV-2 using CaverDock and machine learning

**DOI:** 10.1016/j.csbj.2021.05.043

**Published:** 2021-05-26

**Authors:** Gaspar P. Pinto, Ondrej Vavra, Sergio M. Marques, Jiri Filipovic, David Bednar, Jiri Damborsky

**Affiliations:** a Loschmidt Laboratories, Department of Experimental Biology and RECETOX, Faculty of Science, Masaryk University, Brno, Czech Republic; b International Clinical Research Centre, St. Ann’s Hospital, Brno, Czech Republic; c Institute of Computer Science, Masaryk University, Brno, Czech Republic

**Keywords:** CaverDock, CaverWeb, Protein dynamics, Machine learning, Tunnel, Virtual screening

## Abstract

The new severe acute respiratory syndrome coronavirus 2 (SARS-CoV-2) causes pathological pulmonary symptoms. Most efforts to develop vaccines and drugs against this virus target the spike glycoprotein, particularly its S1 subunit, which is recognised by angiotensin-converting enzyme 2. Here we use the *in-house* developed tool CaverDock to perform virtual screening against spike glycoprotein using a cryogenic electron microscopy structure (PDB-ID: 6VXX) and the representative structures of five most populated clusters from a previously published molecular dynamics simulation. The dataset of ligands was obtained from the ZINC database and consists of drugs approved for clinical use worldwide. Trajectories for the passage of individual drugs through the tunnel of the spike glycoprotein homotrimer, their binding energies within the tunnel, and the duration of their contacts with the trimer’s three subunits were computed for the full dataset. Multivariate statistical methods were then used to establish structure-activity relationships and select top candidate for movement inhibition. This new protocol for the rapid screening of globally approved drugs (4359 ligands) in a multi-state protein structure (6 states) showed high robustness in the rate of finished calculations. The protocol is universal and can be applied to any target protein with an experimental tertiary structure containing protein tunnels or channels. The protocol will be implemented in the next version of CaverWeb (https://loschmidt.chemi.muni.cz/caverweb/) to make it accessible to the wider scientific community.

## Graphical abstract

**Figure:**
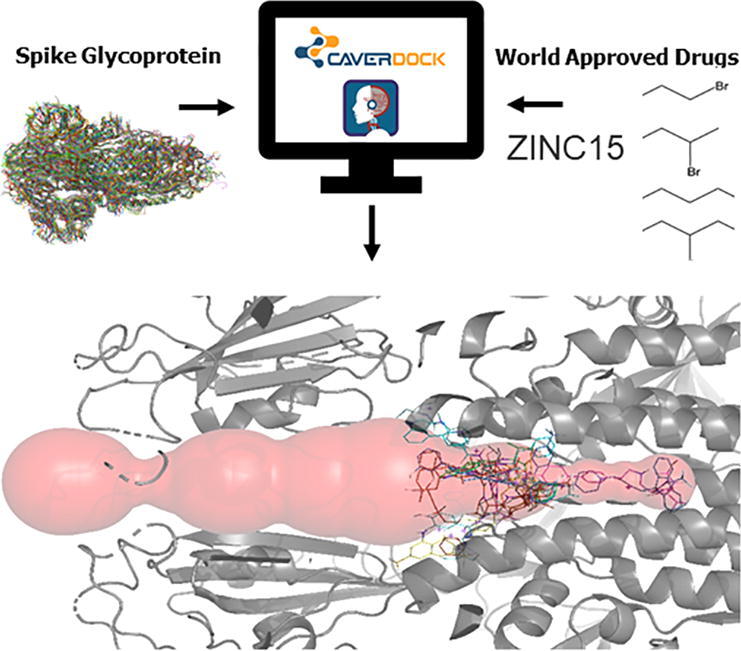

## 1 Introduction

A new coronavirus (SARS-CoV-2) outbreak began in Wuhan in the province of Hubei at the end of 2019. Despite many similarities to the 2002 outbreak of SARS-CoV, the new SARS-CoV-2 outbreak had higher morbidity and mortality. Most infected individuals show mild or no symptoms, but some present general complications such as acute respiratory distress syndrome, pneumonia, and septic shock, potentially leading to the patient’s death [1], [2], [3], [4]. Drawing on established knowledge about the original virus, research groups worldwide have focused their efforts on two viral proteins: i) the spike (s)-glycoprotein, with the aim of disrupting its recognition of the membrane-bound angiotensin-converting enzyme 2 (ACE-2); and ii) the main viral protease (Mpro, 3CLpro) [5], [6], to disrupt viral replication by hindering the processing of several polyproteins that are translated from the viral RNA. Another approach for tackling the spread of the new virus builds on work on the original SARS virus, which resulted in the development of a vaccine designed to induce the production of antibodies against the viral s-glycoprotein [7], [8], preventing it from recognising and binding to ACE-2. Unfortunately, at the time [9], work on this vaccine was discontinued because it had side effects in animal models that prevented its testing in humans [10], [11].

Even though there are already several vaccines in the market for the prevention of SARS-CoV-2 [12], [13], [14], [15], [16], there are currently over 300 therapies [17], [18], [19], [20] in development that are intended to prevent the spread of the virus (https://covid-19tracker.milkeninstitute.org/) and minimize side-effects [21], [22], [23], [24]. These efforts to create a vaccine or a potent inhibitor that can be used as an a *posteriori* medical treatment with acceptable side-effects are being undertaken by both private companies and academic institutions. Both viral and host proteins are being targeted. While most efforts are focused on disrupting the viral protease or viral polymerase, the viral genome is also being targeted to disrupt its replication. In particular, the host enzymes involved in nucleotide synthesis are being studied to halt the final step in viral genome replication. However, most therapies in development target proteins acting upstream of replication; there are almost 40 preclinical and over 30 clinical trials targeting viral surface proteins including the s-glycoprotein. Several host cell membrane proteins are also being targeted, including CD147 and TMPRSS2 [25] and, most importantly, ACE-2 [26], [27].

When the SARS-CoV-2 enters the body, s-glycoprotein units on the surface of the virus act as “hooks”, triggering attachment to a host cell [28], [29], [30]. The s-glycoprotein is homo-trimer with three domains—the cytoplasmic tail, the transmembrane region, and the ectodomain [31]. The ectodomain is further divided into three areas: the proximal membrane region, the S2 subunit, and the S1 subunit. The receptor-binding domain is located in the S1 subunit. ACE-2 recognises the S1 subunit, and between 1 and 3s-glycoprotein monomers can bind to ACE-2 by opening and moving upwards. The covalent bond between subunits S1 and S2 is primed for cleavage to permit the displacement of the S1 subunit before the s-glycoprotein/ACE-2 binding event. The viral membrane then fuses with that of the host cell via a series of substantial conformational changes. Blocking these conformational changes would be a way to taper the propagation of the virus [32]. There are published studies, which targeted in this way HIV protease [33] and the s-glycoprotein of both the SARS-CoV [34], [35] and MERS-CoV viruses [36].

Several conformations of the viral s-glycoprotein have been observed by electron microscopy, including both semi-open (PDB ID 6VYB) and closed (PDB ID 6VXX) conformations [37]. The existence of visibly different conformations demonstrates that the viral s-glycoprotein can undergo conformational changes affecting not just its surface but also the gorge within the S1 subunit and the S2 subunit. Previous docking and virtual screening studies have focused on localised sites such as the active site of the viral Mpro protease [38], [39], [40], [41] or the receptor-binding domain of the s-glycoprotein [42], [43], [44]. There were also studies aiming at drug repurposing targeting the gorge of the s-glycoprotein [45], [46], [47]. A long tunnel created by the formation of the s-glycoprotein trimer has received less attention. Therefore, we decided to search for the drugs that bind in the gorge as well as along the putative tunnel of the ectodomain up to the cleavage site. Studying drug interactions in such long tunnels would be laborious and computationally expensive if using alchemical [48], [49] or ligand migration methods [50], [51]. A long tunnel in a dynamical protein is a perfect target for study using the software tool CaverDock [52], [53], [54].

CaverDock is an *in-house* tool that uses Caver [55], to identify tunnels in protein structures, and an optimised version of the well-established algorithm from AutoDock Vina to calculate possible ligand trajectories along those tunnels and the corresponding binding energies [56]. CaverDock discretises each identified tunnel into a series of discs and models a ligand’s passage through the tunnel by constraining one ligand atom to lie within a disc, sequentially. The ligand’s conformation and binding energies are then calculated using Autodock Vina, with the ligand (aside from the constrained atom) being free to explore the conformational space; the protein is treated as a rigid body. Once the conformation and binding energy have been calculated, the constrained atom is shifted to the next disc and the process is repeated until the ligand has moved through the full length of the tunnel. The tool is continuously maintained and is freely available as both a stand-alone program and a webtool named CaverWeb [57], [58].

Since the start of the pandemic, the scientific community has recognized the need for collaboration and sharing of results by pledging to make data publicly available as soon as possible. In this work, we used data from a 10 µs molecular dynamics (MD) simulation of the s-glycoprotein trimer conducted at the D.E. Shaw Institute [59], from which we extracted the main representative conformations. We also used the original closed structure of the s-glycoprotein retrieved from the Protein Data Bank, giving a total of six structures to study [60]. Each structure was subjected to virtual screening using every drug in the globally approved drugs subset of the ZINC15 database [61]. This subset contained at the time of retrieval, 4359 unique drugs approved by the US Food and Drug Administration, European Medicines Agency, and other significant authorities. A single drug in the subset was not correctly handled by MGL tools for lack of parameters and the virtual screening was done with 4358 unique drugs.

Although the MD simulation that we used is remarkably long by almost any standards (10 µs), all the conformations used in this study came from a single simulation, except the structure obtained by cryo-EM. A better assessment of the full canonical ensemble could be obtained by performing several replicas in the simplest scenario. Even more complex and comprehensive sampling could be achieved by using enhanced sampling methods [62], for example adaptive sampling [63], umbrella sampling, [64] metadynamics, [65] replica exchange molecular dynamics [66] and others [67], [68], [69]. Although for smaller proteins this could be achievable in a reasonable amount of time, for proteins as large as the s-glycoprotein (1353 residues) such task becomes very time demanding and computationally expensive.

**Fig. 1:**
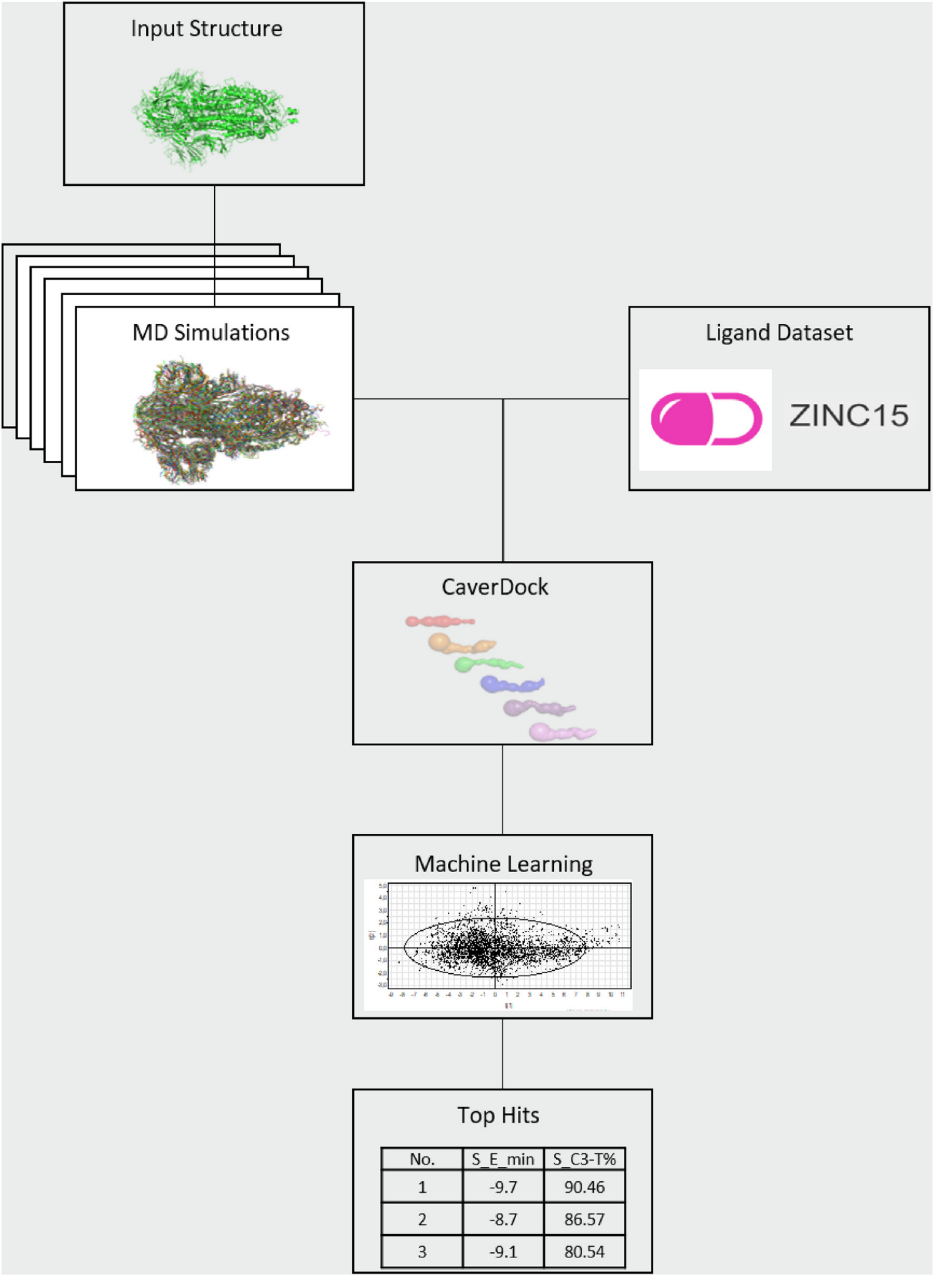
Computational workflow showing the steps performed during the virtual screening with CaverDock using the full globally approved drug dataset and six protein states, along with the subsequent analytical steps. This workflow is currently being implemented on the web server CaverWeb [29] to allow the wider community to easily perform such virtual screens.

Binding energies along the s-glycoprotein tunnel were calculated for every drug and all six structures. We then compared the results obtained to identify the best ligands for each tunnel position in each conformation. We also analysed each drug to identify the contacts made with each monomeric unit of the s-glycoprotein trimer. This allowed us to select drugs that were predicted to interact with all three monomers and are thus likely to suppress the opening of the S1 subunits and thereby prevent the binding of the s-glycoprotein to ACE-2. Quantitative structure–activity relationships analysis (QSAR) was carried out to correlate the binding energies of the drugs with their physicochemical properties using multivariate statistical methods, providing the top-scoring molecules based on their interactions with individual conformations of s-glycoprotein (Fig. 1). The computational workflow established within this study can be generalized and automated to make it applicable to other target proteins.

## 2 Methods

### 2.1 Construction of the s-glycoprotein ensemble

The cryo-EM structure of the trimeric SARS-CoV-2 spike glycoprotein was obtained from the RCSB Protein Data Bank [70]. The selected structure (PDB ID: 6VXX) corresponds to the closed state of this protein. To obtain sufficient conformational diversity for our analysis of the s-glycoprotein trimer, we used the results of a 10 µs MD simulation conducted by the D. E. Shaw group, which started from the same cryo-EM structure of s-glycoprotein. This trajectory was clustered using the cpptraj [71] module of AmberTools 16 [72] and a distance-based metric defined by the mass-weighted root-mean-square deviation (RMSD) of the backbone atoms of the residues surrounding the gorge of the S1 domain. The RMSD was calculated relative to the starting structure. All residues located within 20 Å of the centreline of the tunnel in the initial s-glycoprotein structure (calculated as described below; 565 in total) were included when calculating this metric. The hierarchical agglomerative clustering algorithm was used with average-linkage, a minimum distance between clusters (epsilon) cut-off of 2.5, sieve 5, and a minimum of 5 clusters.

### 2.2 Tunnel analysis

Before the tunnel analysis, three protein segments were removed from the MD snapshots (residues 365 to 372, 1333 to 1340, and 2301 to 2308 – equivalent to residues 447–470 in chains A, B and C in the cryo-EM structure numeration (PDB ID: 6VXX). These segments were detached from the protein during the MD simulation and became unrealistically bound at the mouth of the s-glycoprotein tunnel (see discussion below).. The tunnel extending through the s-glycoprotein trimer was characterized using HOLE v2.2.005 [73]. The vector for the HOLE calculation was defined by the centre points between the C-alpha atoms of the following residues: LYS 1034 and PRO 986 in all three subunits of the s-glycoprotein structure, and LYS 858, 1826, 2794 and PRO 810, 1778, 2746 in the MD snapshots. A sample rate of 0.9 Å was used, and the end radius was set to 10 Å. We analysed the tunnel radii and cut the segment going through the S1 domain until the first extreme tunnel bottleneck was reached; the distance at which this bottleneck was encountered varied between 60 and 80 Å depending on the structure or snapshot under consideration. The output of the HOLE was converted into the CAVER 3 PDB file format [55] to enable discretization for CaverDock calculations. However, the tunnel predicted by HOLE for the s-glycoprotein structure contained disconnections that made it undiscretisable. Therefore, we re-modelled this tunnel using CAVER 3.02, starting from C-alpha of Thr A 1009. The probe radius, shell radius, and shell depth were set to 0.7, 20, and 20, respectively. Finally, the selected tunnel parts were discretized into a series of discs using the discretiser tool with default settings [53].

### 2.3 Ligand dataset

The globally approved drug dataset was downloaded from the ZINC database [61] on the 26th of May 2020 in mol2 format. Only the first protonation state of each drug molecule was saved. The SMILES codes for all ligands were collected and stored in CSV files, which were then uploaded to the Mordred [74] web server to obtain the molecular descriptor values needed for the QSAR calculations.

### 2.4 CaverDock calculations

Only the part of the tunnel in the S1 domain was considered in the CaverDock calculations. We discretised the tunnel into a set of discs using the program’s default settings [53]. The ligand and receptor files were prepared using MGLtools 1.5.7 [75]. The grid box was generated around the relevant part of the tunnel using a script from the CaverDock package. The default drag atom (i.e. the atom closest to the centroid of the molecule) was used. Calculations were run in the inward direction only, in the lower-bound trajectory mode.

### 2.5 Principal components analysis

Principal Component Analysis (PCA) [76] was used to facilitate understanding of the data resulting from the CaverDock calculations. The data matrix consisted of 4358 ligands (objects) docked into six different protein states obtained from the CaverDock trajectories. The data for each ligand consisted of its minimum binding energy along the CaverDock trajectory and three percentage values representing the proportion of the trajectory during which the ligand was in contact with one, two, or all three individual units of the s-glycoprotein trimer. The data were autoscaled to unit variance and centred before analysis.

### 2.6 Partial least squares analysis

Partial Least Squares (PLS) analysis [77] was used to explore the relationships between the minimal binding energies of 4358 ligands (objects) docked to six different protein states (dependent variables Y) and 1326 molecular descriptors of individual ligands (independent variables X). 2D and 3D molecular descriptors were calculated using the software tool Mordred [74] which is particularly suitable for our purpose because it can calculate descriptors even for large molecules. PLS reveals the correlation structure among variables X and Y by reweighting variables X with PLS weights and projecting them to a smaller number of new latent variables. Autoscaled and centred data were used in the PLS analysis. The importance of every molecular descriptor in the model was assessed using the variable importance in the projection (VIP) parameter [78] and plots of the PLS variable weights [78]. Internal validation was performed to assess the quality of the developed PLS models [79] by cross-validation and permutation testing. During cross-validation [77], a portion of the Y data are excluded during model development, and the resulting model is used to predict the missing data. The predictions are then compared to the original data to obtain a Q^2^ value. Q^2^ provides a more realistic estimate of a model’s predictive power than the squared multiple regression coefficient R^2^. In this study, 1/7 of the compounds were deleted during each cross-validation round. During permutation testing, the model was recalculated 999 times by randomly re-ordering the dependent variable y. The statistical package SIMCA-P version 12 (Umetrics, Umeå, Sweden) was used to perform all statistical analyses.

### 2.7 MM/GBSA calculations

The free energy of binding (ΔG_bind_) was calculated by the molecular mechanics/generalized Born solvent accessible surface area (MM/GBSA) method [80], [81] to determine the interaction energy of each drug bound to the spike glycoprotein in the minimum-energy snapshots for each CaverDock calculation with the 6VXX structure. The topology and input files were prepared for each complex for performing an energy-minimization cycle and the energy calculations. The atomic partial charges of each ligand were obtained from the ZINC data base as MOL2 files, and converted to the PREPI files and the parameter modification files (*frcmod*) using the *Antechamber* module of AmberTools 14 [82]. The tLEAP program of AmberTools 14 was then used to specify the ff14SB force field [83], the parameters for the ligands and the Born radii as *mbondi3*. The complexes were minimized using the PMEMD.CUDA [84], [85] module of AMBER 16 [86] due to the large size of the systems. Five rounds of optimization were conducted in an implicit generalized Born solvent (*igb = 8*), each one consisting of 2500 cycles of steepest descent followed by 7500 conjugate gradient cycles, were performed as: (i) one step with all heavy atoms restrained with 500 kcal/mol∙Å^2^ harmonic force constant, and (ii) four steps with decreasing restraints on the protein backbone atoms with 500, 125, 25 and 1 kcal/mol∙Å^2^ force constant. The cut-off for the non-bonded interactions was set to 1000 Å. The a*nte-MMPBSA.py* [80] module of AmberTools 14 was used to convert the original topology of the complex and specify the Born radii as *mbondi3*, and generate the corresponding topology files for the *complex*, *receptor* and *ligand*, to be used in the MM/GBSA calculations. The *MMPBSA.py* [80] module of AmberTools 14 was used to calculate the free energy of binding between the protein and the ligand in the complex after the minimization cycle. The generalized Born method was used (*&gb namelist*) with implicit generalized Born solvent model (*igb = 8*) and 0.1 M ionic strength (*saltcon = 0.1*). The solvent accessible surface area was computed with the LCPO algorithm [87]. Decomposition of the pairwise interactions was generated *(&decomp* namelist), with discrimination of all types of energy contributions (*idecomp = 4*) for the whole residues (*dec_verbose = 0*).

### 2.8 Analysing the similarity between the top ten hits

We analysed the 10 best binders in order to identify which common structural features could be used as a basis for searching similar drugs in the future. We performed this structural search using the FindMCS module from RDKit 2016.03.5 [*https://github.com/rdkit/rdkit*]. The parameter ringMatchesRingOnly was switched to True, so that the aliphatic carbon chains would not be matched with aromatic rings. We analysed all ten molecules and every pair combination. Furthermore, we calculated the Tanimoto similarity with the DataStructs.FingerprintSimilarity module to quantify the similarity of molecules in each pair.

## 3 Results and discussion

### 3.1 Cryo-EM structure of spike glycoprotein

We initially analysed the cryogenic electron microscopy (cryo-EM) structure in the closed conformation (PDB ID: 6XVV). This choice was made because our objective was to block the viral infection mechanism by over-stabilizing the closed conformation to suppress the protein’s biological activity. Despite missing some loops on the surface, the cryo-EM structure had a sufficiently high resolution and structural integrity inside the tunnel for virtual screening with CaverDock. Because the goal was to block large conformational changes of the s-glycoprotein trimer, we ranked the best binding drugs based on both their overall binding energies and the extent of their contacts with all three monomeric units. Three distinct clusters of drugs with binding profiles showing clear energy minima were identified, each binding to a different region of the tunnel (Fig. 3). The first cluster consisted of drugs binding in the region immediately behind the first bottleneck of the subunit S1 gorge, between 12 Å and 21 Å from the trimer’s surface. Since this region is immediately behind the tunnel’s second tightest bottleneck, we hypothesise that drugs in this cluster are flexible enough to cross that narrow part of the tunnel and then undergo a conformational change to adopt an optimal binding conformation.

**Fig. 2:**
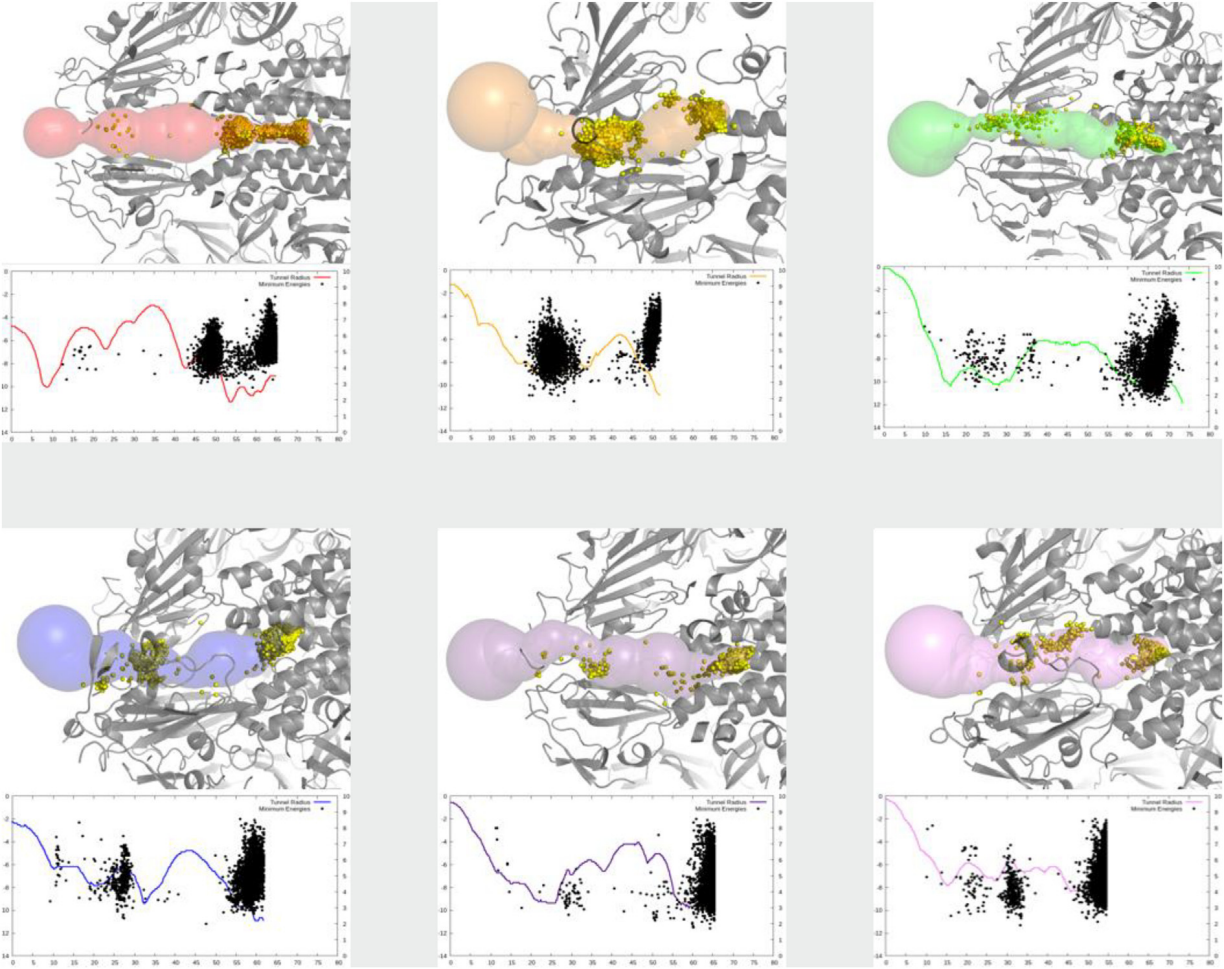
Tunnels in the six protein states showing the regions where the drugs bind with the lowest binding energy. Top: Visualization of the tunnel used for virtual screening in the six protein states analysed with CaverDock. These states are the cryo-EM structure (red) and 5 representative structures (s1 in orange, s2 in green, s3 in blue, s4 in purple and s5 in pink) obtained by clustering the results of an MD simulation. Yellow spheres in the tunnels indicate the centre of mass of each drug when bound at the location where it binds most strongly. The plots below each structure show the corresponding tunnel profiles (in Å) using solid lines. Each black dot indicates the position where one drug binds most strongly together with the corresponding binding energy in kcal/mol. (For interpretation of the references to colour in this figure legend, the reader is referred to the web version of this article.)

The second and smallest cluster of drugs binds in the middle of the tunnel. Although we consider this group to be a cluster, the binding positions of the drugs at the extremes of the cluster differ by 10 Å: ZINC000004099004 binds 26 Å from the surface, while ZINC000008214470 binds at 36 Å. The final region of the tunnel is also the most populated; 99.5% of the drugs tested in the virtual screen bind most strongly in its deepest third, between 45 Å and 65 Å from the surface. All the top ten drugs identified in this study (Fig. 2) belong to this final cluster and have consistently lower binding energies than any drug binding preferentially in the other two regions. Besides, most of the drugs with the lowest binding energies belong to the cluster binding at the position 3 (Electronic Supplementary Information ESI – Energy plots folder (https://loschmidt.chemi.muni.cz/data/caverdock/pinto_2021_suppl/). The profile of the tunnel in this region is narrower than in the other tunnel regions.

**Fig. 3:**
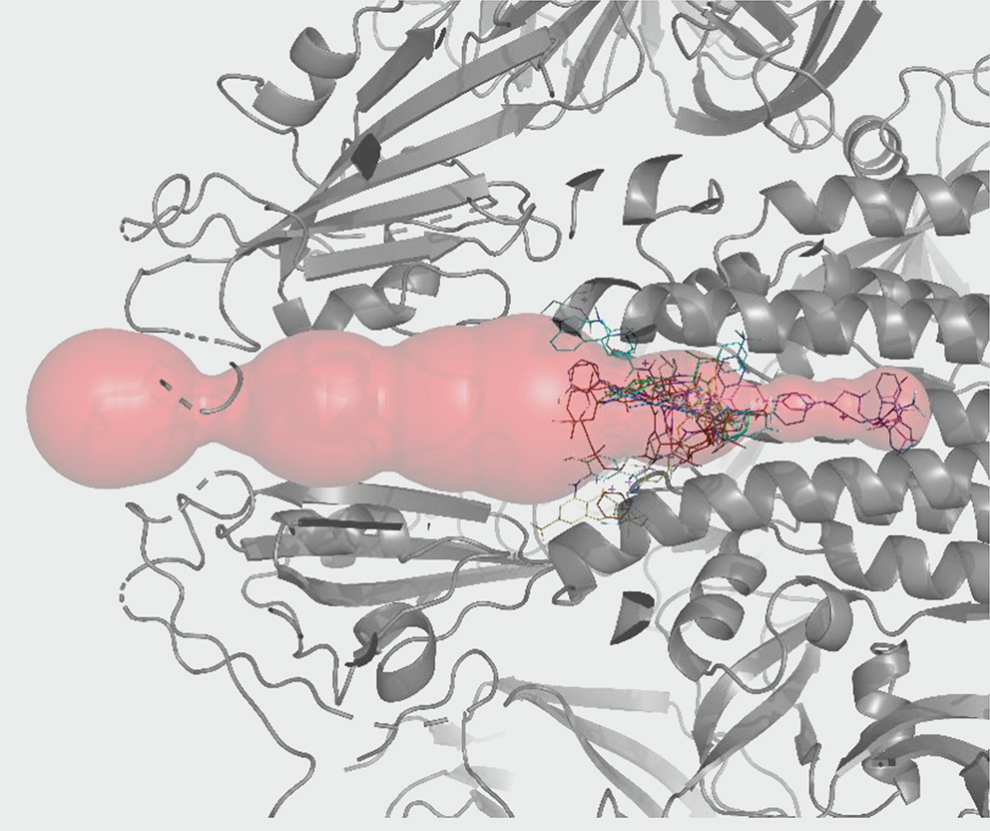
Visualization of the tunnel in the cryo-EM structure with the top ten inhibitors bound to the positions corresponding to their lowest binding energy. The drugs were ranked by multivariate analyses presented below (Fig. 4). The protein structure (PDB ID: 6VXX) is shown as a grey ribbon, while the tunnel predicted by CaverDock is indicated by the red surface. Inhibitors are shown using all-atom models, coloured by atom type. (For interpretation of the references to colour in this figure legend, the reader is referred to the web version of this article.)

### 3.2 The s-glycoprotein dynamical ensemble

The D. E. Shaw research institute studied the dynamical ensemble of the s-glycoprotein by performing a 10 µs MD simulation starting from the closed cryo-EM structure mentioned above (PDB ID: 6VXX). This simulation became stable after 6 µs, as shown by the root-mean-square deviation (RMDS) plot (SI-Fig. 1). Due to the s-glycoprotein’s high flexibility, the cryo-EM structure lacks several parts of its sequence, causing several segments to be seemingly disconnected from the rest of the structure. Unfortunately, during the MD simulation, two fragments corresponding to residues 447–470 in chains A, B and C in the cryo-EM structure numeration (PDB ID: 6VXX) detached themselves from their correct positions and drifted to different locations within the structure. These events are responsible for the two spikes seen in the RMSD plots at around 2.1 and 5.2 µs (SI-Fig. 2). These unrealistically dynamical fragments, which were originally located on the outer surface of the s-glycoprotein were excluded from all subsequent analyses in this work.

We clustered the MD snapshots based on the RMSD of the gorge residues to obtain diverse but biologically relevant conformations of the s-glycoprotein. The obtained clusters are ranked in terms of their populations. The most populated cluster, s1, dominated almost the entire second half of the trajectory (SI-Fig. 1). The mean RMSD of the gorge residues in this cluster was 3.46 ± 0.13 Å, which is close to the average value for the entire simulation (3.66 ± 0.38 Å) (SI-Fig. 3). Conversely, the least populated cluster (s7) had RMSD values indicating that it remained close to its starting structure (1.62 ± 0.69 Å). Representative structures of the clusters (SI-Fig. 3) and their tunnels (SI-Figs. 5 and 6) were also obtained, enabling further analysis (SI-Fig. 3).

CaverDock calculations were performed using representative structures of the 5 most populated clusters in the same way as described for the cryo-EM structure (Fig. 2). Each tunnel had a unique profile, but in all cases, the narrowest section was in the deepest region of the tunnel, close to the S2 subunit. The vast majority of the ligands have their lowest binding energies in this region (Fig. 3). This was expected given that this region resembles a binding pocket with many possible molecular interactions. The sole exception is the most populated state, s1, for which the majority of the ligands have their lowest binding energies in the middle of the tunnel (Fig. 2). The tunnel in this state is slightly wider than in the other states, making it difficult for ligands to form contacts with all three monomers. The tendency for the binding energies of drugs to be lowest immediately before or after a bottleneck was seen for all states.

### 3.3 Principal Component analysis (PCA)

Multivariate statistical analyses were used to: (i) comprehend the large data sets obtained from the CaverDock calculations, (ii) establish structure–activity relationships, and (iii) select the best potential drug candidates. Two statistically significant models were generated by PCA using the CaverDock results obtained using the set of 4358 ligands and six protein states. The data used in the PCA were the minimum binding energies for each drug along the trajectory and the proportions of the trajectory during which the docked ligand was in contact with one, two, and all three individual subunits of the s-glycoprotein trimer, expressed as percentages.

**Fig. 4:**
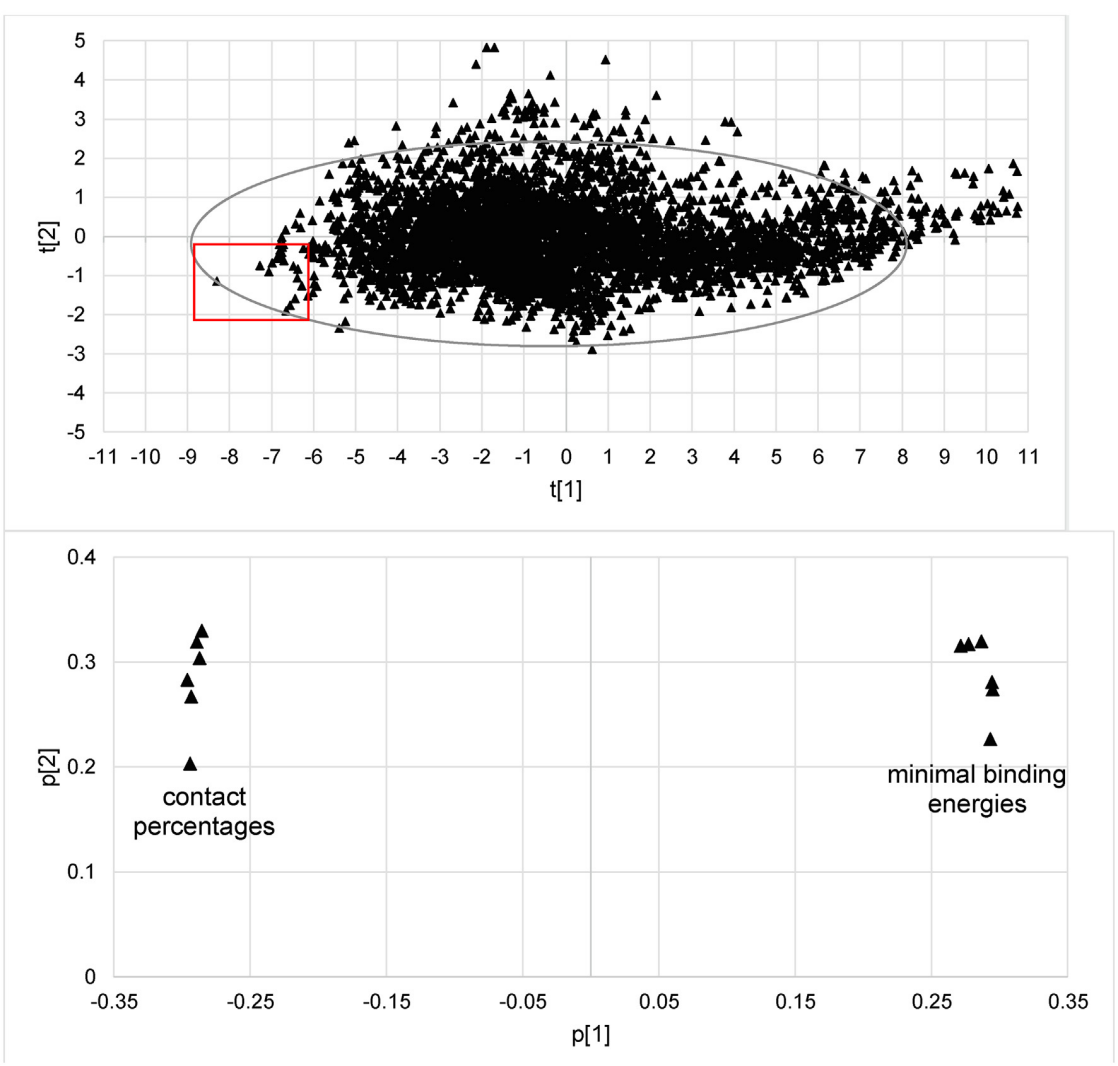
Scores and loadings plots of the first two principal components of the second PCA model. Top: Scores plot of the first two principal components showing the distributions of all studied compounds based on their minimal binding energies and number of contacts with the three subunits of the spike glycoprotein. The top hits were selected from this plot. The positions of the compounds in the 2D space are determined by the locations of variables in the loadings plot (bottom). Compounds showing the strongest binding to all three units in the different states of the spike protein are located on the left of the plot (red box). Bottom: Loadings plot of the first two principal components showing the distribution of the variables in the 2D space. This plot corresponds to the scores plot presented above. The variables describing the minimal binding energies calculated for the six different s-glycoprotein states are on the right, while those describing the contact percentage with the three individual subunits of the spike protein trimer are located on the left. (For interpretation of the references to colour in this figure legend, the reader is referred to the web version of this article.)

The first PCA model (PCA-1) used 24 variables: 3 related to the minimum binding energies for each protein state, and 3 quantifying the percentages of the trajectory during which the drug was in contact with 1, 2, and 3 units of the trimeric s-glycoprotein. Ten statistically significant principal components were obtained, collectively explaining 98% of the variation in the data. The second model, PCA-2, was generated using 12 variables representing the energy minima and the percentages of each trajectory during which the drug was in contact with all three monomeric units of the s-glycoprotein trimer for each of the six studied protein states. This model yielded only two principal components that explained 85% and 8% of the variation in the data, respectively. Because it had only two principal components, this model was easier to interpret than the first. The top hits predicted by the two models were very similar, so only the results obtained with the simpler model 2 will be discussed further. By inspecting the distribution of the docked compounds in the 2D space spanned by the first two principal components (Fig. 4), the compounds interacting most strongly with all three subunits of the spike protein were identified (ESI - pml_sessions/session-6vxx). Such compounds are most likely to modify the conformational behaviour of the s-glycoprotein and thus affect its biological function. The distribution of the 12 variables used to cluster the ligands is shown at the bottom of Fig. 4.

### 3.4 Partial least squares analysis (PLS)

A PLS analysis was performed to correlate the minimum binding energies for each ligand from the CaverDock calculations with the molecular descriptors of the docked ligands. Binding energies calculated for all six states of the s-glycoprotein were considered simultaneously using a single PLS model. The initial model, PLS-1, used 1326 independent variables and consisted of four principal components collectively explaining 87% of the variation in the data. The correlation coefficient (R^2^ = 0.87) and cross-validated correlation coefficient (Q^2^ = 0.87) of this model are identical, suggesting excellent predictive power. To simplify the model, the variable selection was performed. Specifically, independent variables were selected based on their position in the loadings plot and variable importance in the projection (VIP) plot. In this way, the number of variables was reduced from 1326 to 56. A new model generated with these variables, PLS-2, had three principal components, with an R^2^ of 0.84 and a Q^2^ of 0.84. Validation by permutation testing - scrambling the Y variables while keeping the X-matrix unchanged – indicated that this correlation would be very unlikely to be observed by chance, as expected given the large number of observations on which the model is based. The established PLS models are applicable for predictive purposes. The predictions can be made even for extensive sets of compounds and can guide selection of suitable candidates for experimental testing. The PLS models allow prediction of minimum binding energies solely from the molecular structure of the ligands. Molecular descriptors can be generated using the on-line version of MORDRED and inserted as the variables to the model for fast prediction of binding energies.

The observed minimal binding energies were plotted against the corresponding predicted values for the starting structure 6VXX and state s4, for which the worst and best fits were obtained, respectively (SI-Fig. 7). VIP values were computed to quantify the relative importance of the chosen molecular descriptors in explaining the differences in the minimum binding energies for all six states (SI-Fig. 8). The most influential variables were FMF (a molecular framework ratio descriptor of the shape of the molecule), BalabanJ (Balaban’s J graph index, which describes the molecular structure of small molecules), piPC (a path count descriptor of molecular topology), MWC and SRW04 (walk count descriptors, the latter of which relates to self-returning walks), VR (a normalised Randic-like eigenvector-based index derived from the Barysz matrix, weighted by atomic number), and VE (the average coefficients of the last eigenvectors of the Barysz matrix, weighted by van der Waals’ volume). Detailed information about all molecular descriptors computed using MORDRED is available at https://mordred-descriptor.github.io/documentation/master/descriptors.html and in the book 3D QSAR in Drug Design [39].

### 3.5 Top ranked drugs

**Fig. 5:**
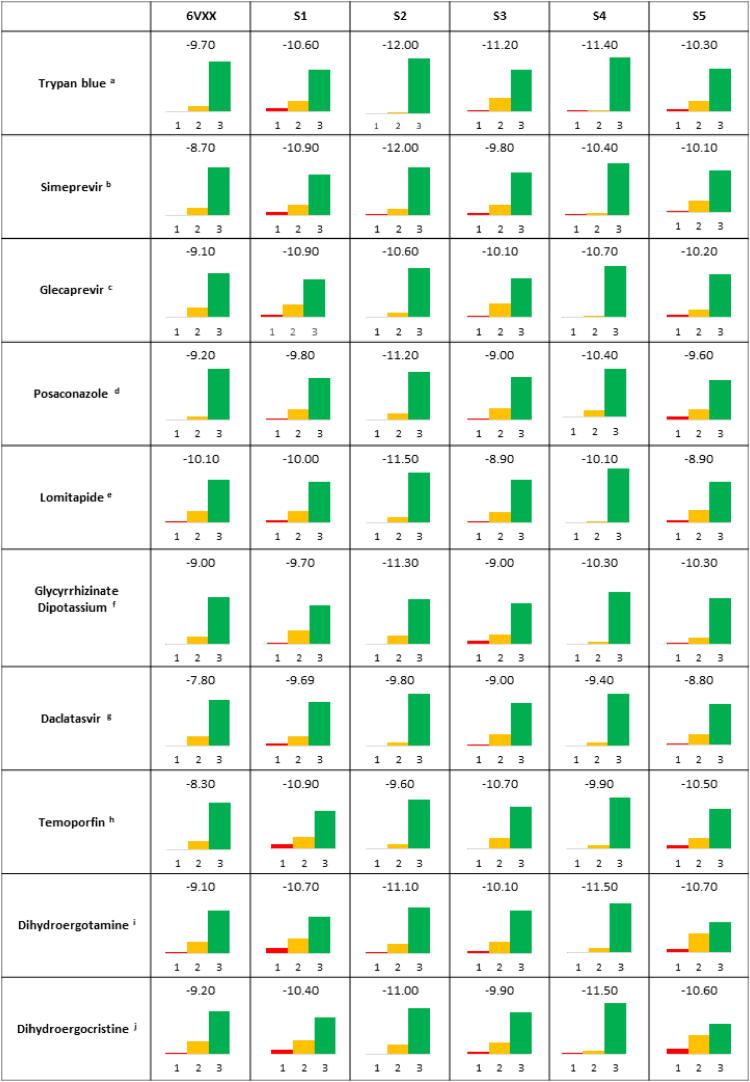

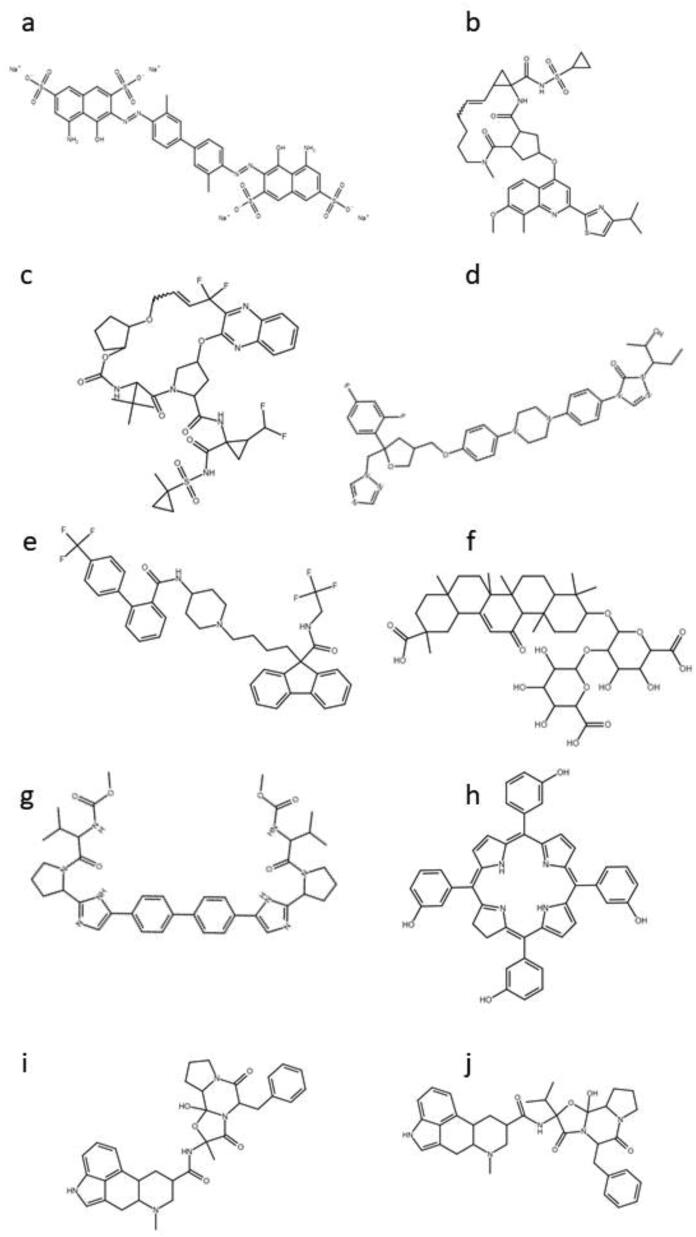
Top ten inhibitors predicted using CaverDock simulations and machine learning. Drug names and labels are shown in the first column; respective chemical structures are shown below the table. Binding energies per drug for each protein state – cryoEM 6VXX and MD states S1-S5 – are reported in kcal/mol. The bar plots under each binding energy represent the percentage of the corresponding trajectory during which these compounds formed contacts with one monomer (red), two monomers (yellow), and three monomers (green). (For interpretation of the references to colour in this figure legend, the reader is referred to the web version of this article.)

We obtained a ranking of the best binders from the PCA and selected the top ten for further analysis (Fig. 5). These ligands had consistently low binding energies in all of the studied protein structures and exhibited a high percentage of contacts with all three monomeric units of the s-glycoprotein trimer during the CaverDock simulations. Although drugs in clusters S1, S3, and S5 occasionally formed contacts with only one monomer, these cases represented less than 10% of the corresponding trajectory. This ranking reflects our assumption that strong interactions with all three monomers in different states of the trimer will reduce the trimer’s capacity for conformational change, which is essential for the biological activity of the spike glycoprotein. We also found that multivariate statistical methods were needed to rank the drugs meaningfully. For example, a simple ranking of the drugs based on their minimum binding energies would not have placed Daclatasvir (Fig. 5) in the top ten because its binding energies for all six conformations are higher than those of some drugs that were not selected. It was thus clear that interaction with all three monomers was weighted strongly in the ranking of the drugs; for three of the studied protein states, Daclatasvir was observed in contact with two and three subunits of the s-glycoprotein trimer, and in the remaining three states (clusters s1, s3 and s5) it was in contact with two or three subunits for at least 96.4% of the trajectory (SI-Table 1).

Among the drugs ranked in the top ten was a dye for cataract surgery (ZINC000169289767), three drugs currently used as antiviral agents against the hepatitis C virus (ZINC000164760756, ZINC000936069565, ZINC000068204830), an antifungal (ZINC000028639340), a microsomal triglyceride transfer protein inhibitor (ZINC000027990463), a hepatoprotective drug for chronic hepatitis (ZINC000096015174), an agent used to treat squamous cell carcinoma of the head and neck (ZINC000003934128), a vasoconstrictor used to treat migraines (ZINC000003978005), and an agent for treating cerebral and peripheral vascular events that are also used in Alzheimer’s studies to inhibit γ-secretase (ZINC000003995616).

The top ten ranked drugs were analysed further. We re-scored the binding energies of the respective drugs with the protein using a very robust, but computationally more demanding method, the molecular mechanics/generalized Born solvent accessible surface area (MM/GBSA) [80], [81]. For this purpose, we took the minimum-energy complexes obtained with CaverDock on the cryo-EM structure, 6VXX. These complexes were minimized using a classical MM force field (AMBER ff14SB^83^), and we calculated the total free energy of binding with the MM/GBSA approach, ΔG_bind_^Total^, as well as the ΔG_bind_ individual contributions from the individual residues. The results showed that most of these drugs can interact with the protein with very strong and favourable energies (SI-Tables 3 and 4). Daclatasvir, Dihydroergocristine and Lomitapide were leading the list in terms of the ΔG_bind_^Total^ values. The only exception was Trypan blue, which showed an unfavourable positive ΔG_bind_^Total^ value in that complex. This suggests the need for larger conformational changes on the protein (with respect to the 6VXX structure) to accommodate this molecule in such a position. If we observe Fig. 5, we find that using CaverDock, Trypan blue scored the best with cluster S2 and not with 6VXX, which is in agreement with our explanation. We recommend prioritizing the experimental testing according to the new ΔG_bind_^Total^ scores (SI-Table 3).

### 3.6 Comparison with previously published virtual screening studies

There were other studies tackling the s-glycoprotein as a whole or its receptor-binding domain RBD (Table 1). Trezza et al. used short classical MD simulations to relax the system and subsequently perform virtual screening with the FDA-approved drugs from Drugbank [88]. Additionally, the authors performed supervised MD simulations to study the binding of the s-glycoprotein RBD to the human angiotensin-converting enzyme 2, and steered MD simulations with two drugs complexed with the RBD of the s-glycoprotein. They present a top ten binding drugs: Lumacaftor, Paritaprevir, Dihydroergotamine, Trypan blue, Midostaurin, Dihydroergotoxine, Simeprevir, Lurasidone, Spinosyn D, and Olaparib. We notice that three of these drugs (Dihydroergotamine, Trypan blue and Simeprevir) were included among top ten hits in our study. Panda et al. performed virtual screening with anti-viral compounds obtained from the ChEMBL database against the three targets: M^pro^, RBD, and s-glycoprotein. The authors then performed MD simulations to validate their best binding drug, pc786, which is still in the clinical trials. This study yielded a top ten for each target, for the RBD they prioritized the drugs: pc786, Tegavivint, Maraviroc, Doxazosin, Dolutegravir, JNJ-49095397, Temsavir, Lorecivivint, VP-14637, and Tecovirimat. On the other hand, following top-ten drugs were selected for the whole s-glycoprotein: pc786, Lorecivivint, Tegavivint, Maraviroc, Itraconazole, Dolutegravir, Troglitazone, Elvitegravir, Danirixin, and Linagliptin.

**Table 1:** Examples of other previously published virtual screening studies that targeted the s-glycoprotein or its receptor-binding domain.

Virtual screening study	Methods	Targets
Trezza et al. [89]	MD simulations	RBD
Panda et al. [90]	Molecular docking; MD simulations	M^pro^; RBD; s-glycoprotein
Kalathiya et al. [91]	Molecular docking; MD simulations	RBD
Wei et al. [92]	Molecular docking	s-glycoprotein
Awad et al. [93]	Molecular docking; MD simulations; MM/GBSA	RBD
Romeo et al. [47]	Molecular docking; MD simulations	s-glycoprotein
Mirabelli et al. [94]	*In-vitro* high throughput assay	Vero E6, Caco-2, LNcaP and Huh7 cells

MD – Molecular dynamics simulations, MM/GBSA – Molecular mechanics with generalised Born and surface area solvation, RBD – Receptor binding domain, M^pro^ – Main protease.

Another virtual screening study targeting the RBD by Kalathiya et al. yielded the drugs that the authors deemed good binders: Chitosan, Rapamycin, Paclitaxel, Selamectin, Everolimus, Ritonavir, and Danoprevir. Wei et al. used the FDA-approved drugs dataset from DrugBank as Trezza’s publication and natural compounds from Traditional Chinese Medicine Systems Pharmacology. They performed virtual screening of these two datasets on RBD and ran short MD simulations on the best-binding drugs. Their top ten binding drugs for the FDA-approved dataset: Digitoxin, Nilotinib, Lemborexant, Raltegravir, Antrafenine, Flunitrazepam, Entrectinib, Flavin adenine dinucleotide, Pazopanib, and Loxapine. Awad et al. studied the same drug dataset. The methodology developed by the authors was however slightly different and yielded different top ten candidates: Silodosin, Ebastine, Salazosulfadimidine, Indacaterol, Chidamide, Regorafenib, Tasosartan, Bagrosin, Lumacaftor, and Risperidone. The authors target the RBD only with classical docking and implemented absorption, distribution, metabolism, and excretion values in their workflow. The authors also used MM/GBSA calculations in order to validate their best binding drugs and suggest eight drugs as promising leads. Romeo et al. targeted the s-glycoprotein with molecular docking and MD simulations in order to confirm the best binding drug’s conformations. Their top ten list of candidate drugs includes: 31 h-phthalocyanine, Hypericin, Dihydroergotamine, JNJ-10311795, TZ2PA6, Quarfloxin, TMC-647055, Tadalafil, and Tepotinib. The drug Dihydroergotamine shows the overlap with our study and Trezza’s study. Another experimental study analysed in depth 17 hits for drug repurposing screening for Covid19 from a library of 1425 43 FDA-approved compounds and clinical candidates. Three of these drugs were not part of our study, as they were investigational, pre-clinical and a dietary supplement. The other 14 were: Amiodarone, Bosutinib, Clofazimine, Domperidone, Entecavir, Fedratinib, Gilteritinib, Ipratropium Bomide, Lomitapide, Metoclopramide, Niclosamide, S1RA, Thioguanine and Verapamil. We conclude that there is an overlap of three drugs (Dihydroergotamine, Trypan blue and Simeprevir) from one study and one drug (Dihydroergotamine) from another study with our results, even though different protocols were employed. Most importantly, there is an overlap of one drug (Lomitapide) with the experimental work done by Mirabelli *et al.* [94], showing that this kind of prioritization should be seen as only that, and not the absolute final data in drug repurposing studies. We stress that the results from theoretical calculations provide prioritization of the potential drugs for the experimental testing but should not be seen as the replacement for the laboratory tests by any means.

### 3.7 Similarity between the top ten binders

Additionally, we have analyzed the most common substructures with the list of our top ten binders using the MCS search implemented in RDKit (SI-Table 5). The analysis revealed that all ten molecules share no common sub-structure. Analysis of all molecule pairs from the data set revealed that the largest common substructure is between Dihydroergotamine and Dihydroergocristine (43 atoms; Tanimoto similarity score 0.98), followed by the pair of Glecaprevir and Dihydroergotamine (15 atoms; Tanimoto similarity score 0.70), and Glecaprevir and Dihydroergocristine (15 atoms; Tanimoto similarity score 0.71). Other pairs showed the common substructures of less than 15 atoms. We report the fingerprints and common substructures in SMARTS format in SI-Table 5.

## 4 Conclusions

Here we describe a computational workflow that was used to perform virtual screening based on CaverDock trajectories for 4358 drug molecules and six conformational states of the s-glycoprotein of SARS-CoV-2. This analysis involved a total of 26,148 calculations. Each calculation took a real-time average of 37 min to complete on 8 CPUs, making the method sufficiently fast for thorough virtual screening. It should be noted that the length of the tunnel in the studied s-glycoprotein structures ranges between 57 Å and 77 Å, making it several times longer than typical enzyme tunnels. However, this long tunnel can serve as a good representative of the structural features present in transmembrane proteins. We used a machine learning to identify the most promising drug candidates based on their strength of binding inside the tunnel and their likely ability to prevent the s-glycoprotein trimer from undergoing functionally necessary conformational change. Although we only selected 10 inhibitors here for the sake of brevity, this number could easily be increased. CaverDock is fast enough to analyse even higher number of snapshots to cover the protein’s conformational space more comprehensively or to examine a significantly greater number of ligands. Importantly, this workflow is currently being made available on the CaverWeb tool to enable automated virtual screenings of the ZINC globally approved drugs dataset. This will enable researchers around the world to perform virtual screening and data analysis in the same way as reported here, in a user-friendly manner. It will also be possible to export the results as comma separated value (CSV) files and/or Pymol sessions to be opened and processed locally by the user. The procedure will be applicable to any protein with an available tertiary structure containing tunnels or channels and should thus find diverse applications in drug design, protein engineering, and metabolic engineering. We are currently implementing this virtual screening platform into CaverWeb [57] to allow the community to perform similar automated calculations against other target proteins using the approved drug datasets.

## Declaration of Competing Interest

The authors declare that they have no known competing financial interests or personal relationships that could have appeared to influence the work reported in this paper.
