# Supplementary material for: Screening of world approved drugs against highly dynamical spike glycoprotein of SARS-CoV-2 using CaverDock and machine learning
Source: Comput Struct Biotechnol J. 2021 May 26;19:3187–97. doi: 10.1016/j.csbj.2021.05.043 (PMC8174816; doi:10.1016/j.csbj.2021.05.043)
Supplement: Supplementary Data 1 [file mmc1.docx]

**SUPPLEMENTARY INFORMATION**

**Screening of World Approved Drugs against Highly Dynamical Spike Glycoprotein SARS-CoV-2 using CaverDock and Machine Learning**

Gaspar P. Pinto,^a,b,†^ Ondrej Vavra,^a,b,†^ Sergio M. Marques,^a,b^ Jiri Filipovic,^c^ David Bednar ^a,b,*^, Jiri Damborsky ^a,b,*^

^a^ Loschmidt Laboratories, Department of Experimental Biology and RECETOX, Faculty of Science, Masaryk University, Brno, Czech Republic; ^b^ International Clinical Research Centre, St. Ann’s Hospital, Brno, Czech Republic; ^c^ Institute of Computer Science, Masaryk University, Brno, Czech Republic

^†^ Authors contributed equally to this work; ^*^ Authors for correspondence: 222755@mail.muni.cz and [jiri@chemi.muni.cz](mailto:jiri@chemi.muni.cz).


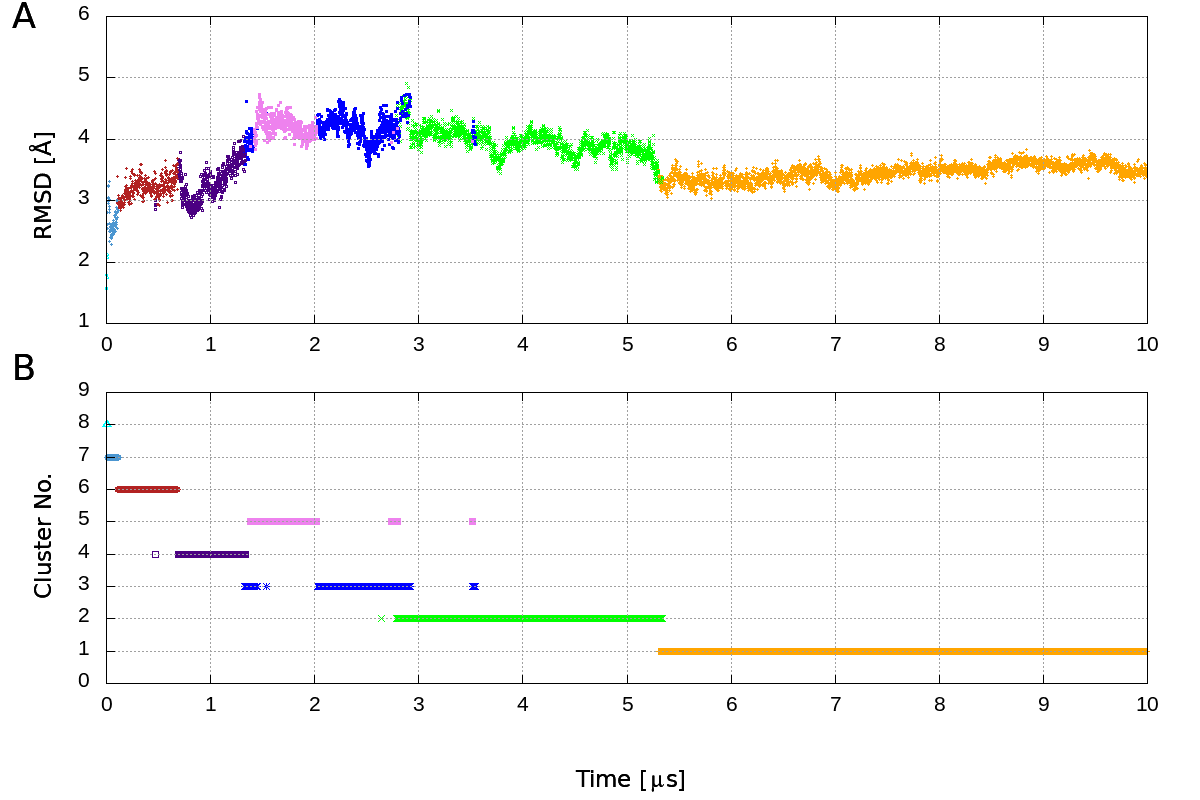


**SI-Figure 1** Clusters obtained from the MD simulations of the spike-glycoprotein. A) variation in time of the RMSD of the gorge residues (calculated for all heavy atoms; used as the metric for clustering the MD), coloured by cluster; B) the clusters’ distribution during the MD. The population of each cluster decreases with the increasing of the cluster number (*cluster 1*, in orange, is the most populated).


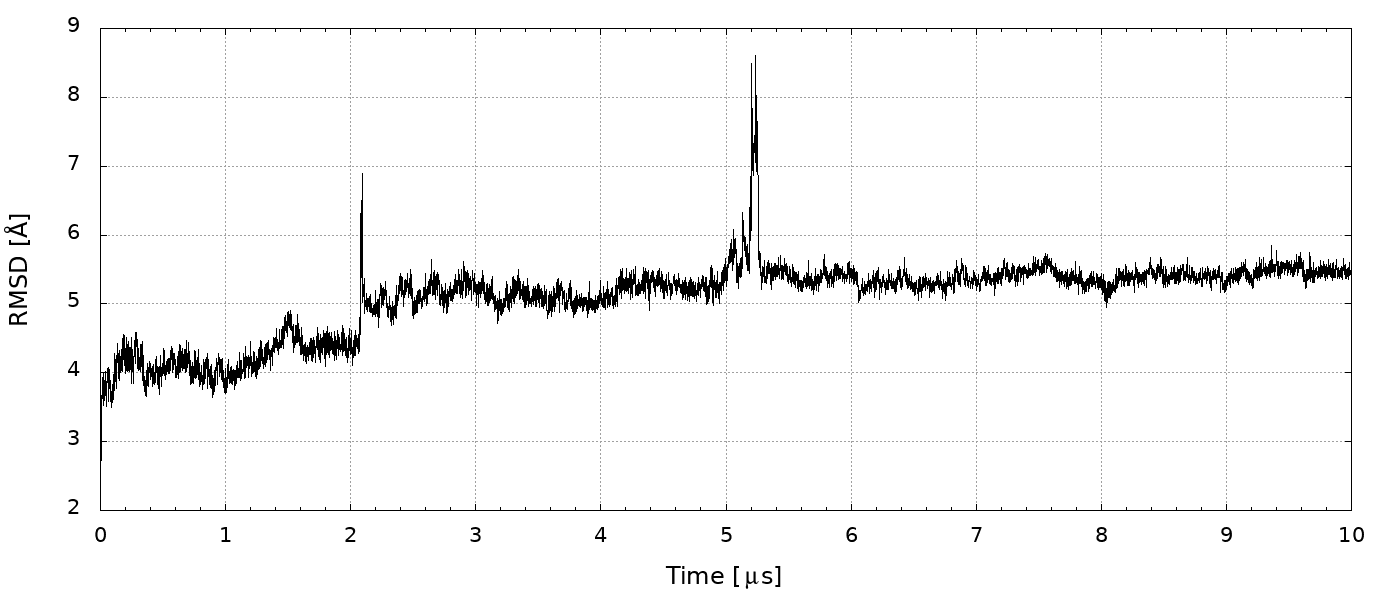


**SI-Figure 2** RMSD of the s-glycoprotein structure during the MD simulation with respect to the starting structure. Values calculated for the backbone atoms.


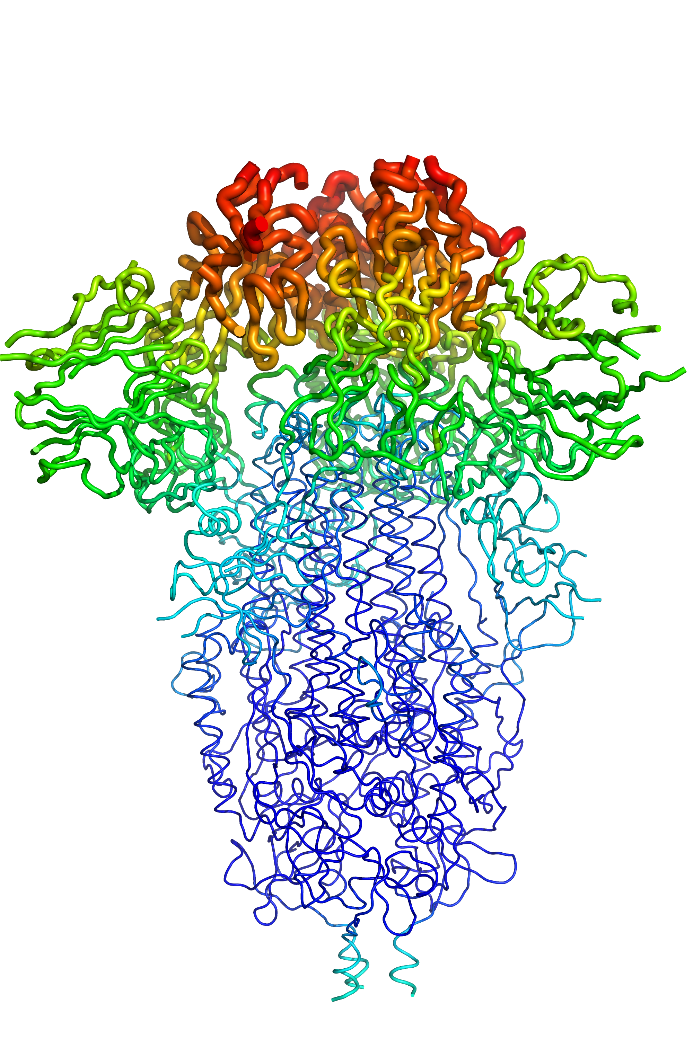

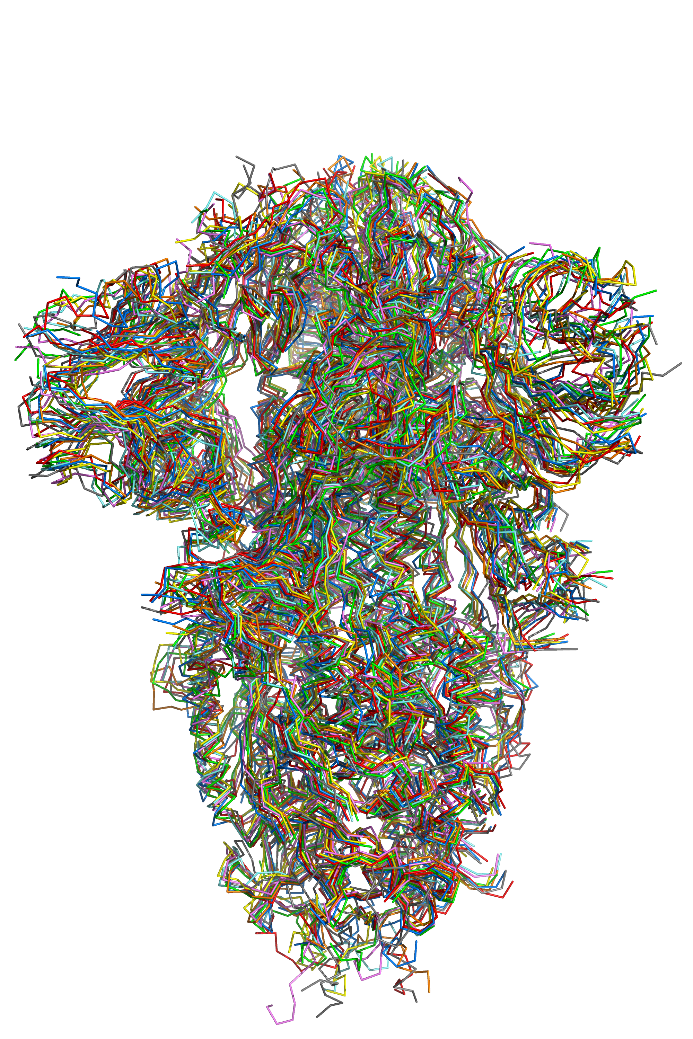


**SI-Figure 3** Global representative structures of the cluster. A) cryo-EM structure showing the B-factors in a putty representation (PDB ID: 6VXX); B) superimposition of all 8 clusters in the same orientation as in A). The cluster structures are displayed as ribbons in the respective colours as in SI-Figure 1.


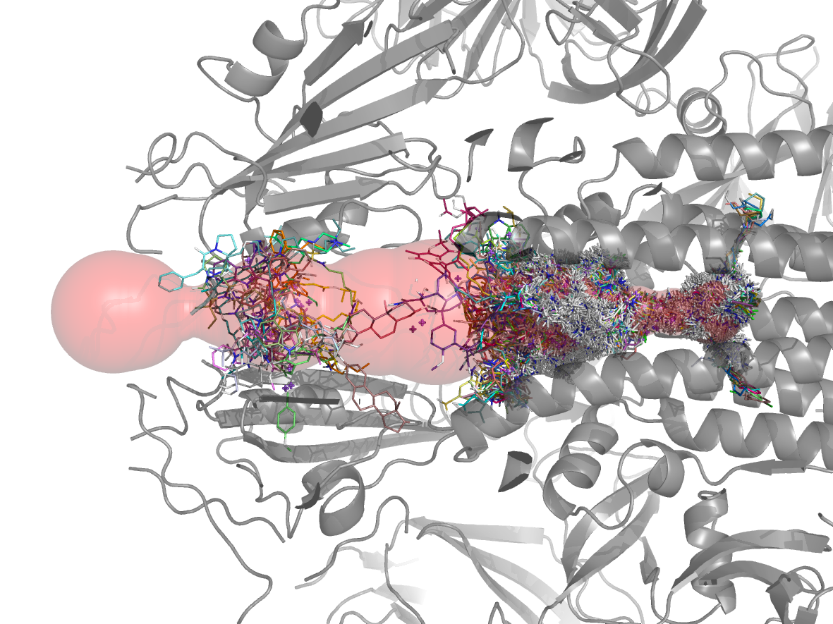


**SI-Figure 4** Cryo-EM structure with the full dataset bound in the tunnel in the place where each drug had their best binding energy. Highly flexible drugs had their best binding energy close to the end of the tunnel where they could bind in between the helices of the protein.


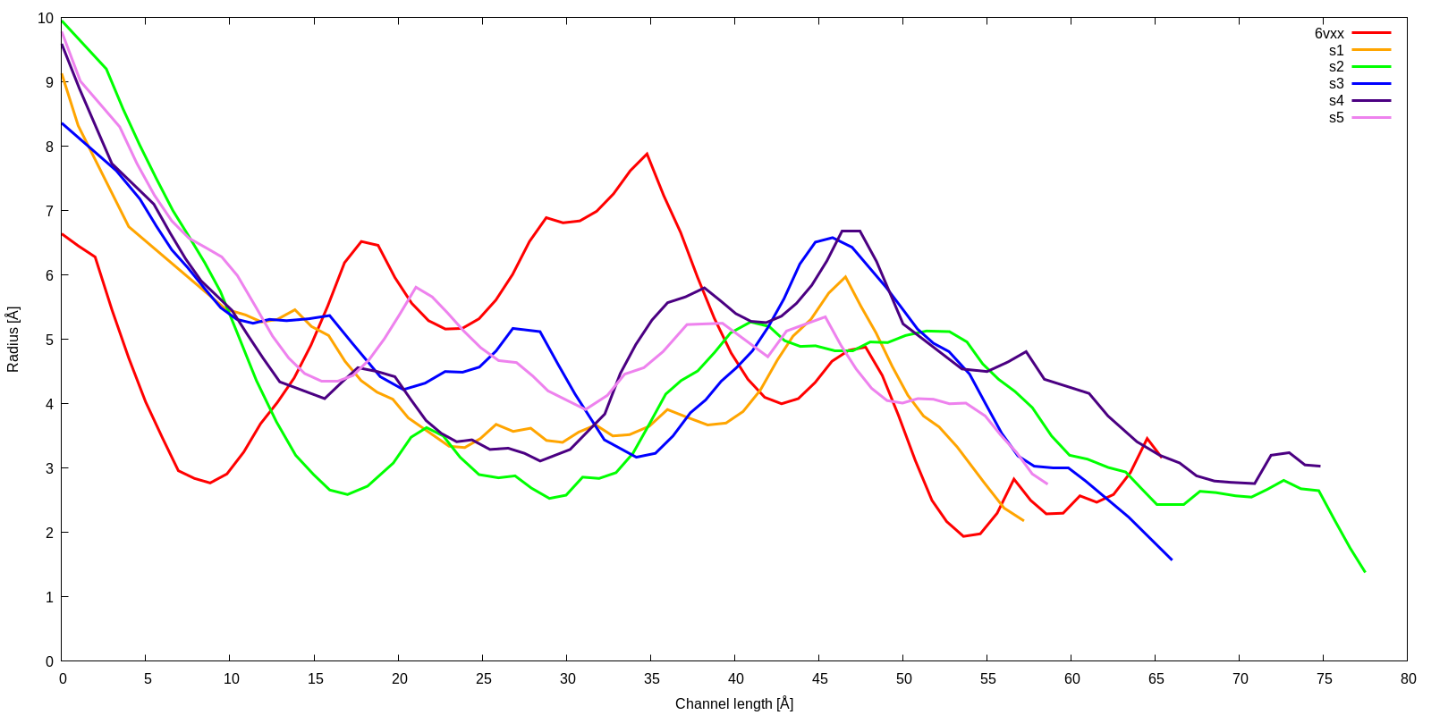


**SI-Figure 5** Comparison of all the tunnel profiles. On the y-axis, we represent the radius of the tunnels and on the x-axis the length of the tunnels. Colour coding is the same as for the previous figures, as shown in the legend on the top right corner of the figure.


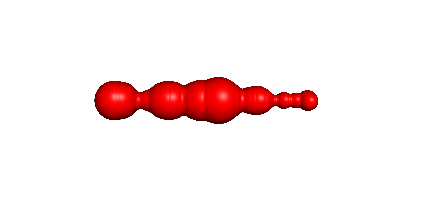

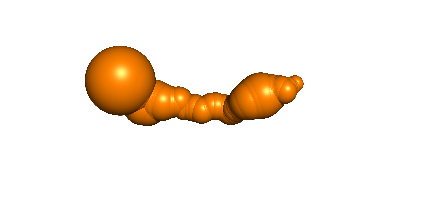

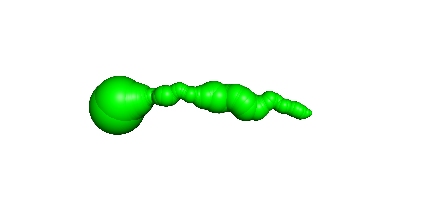

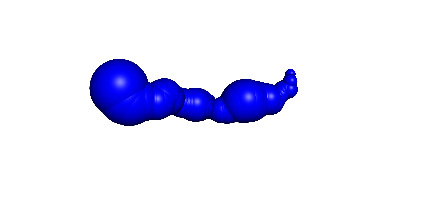

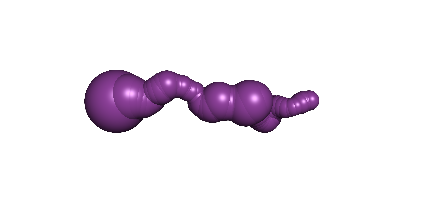

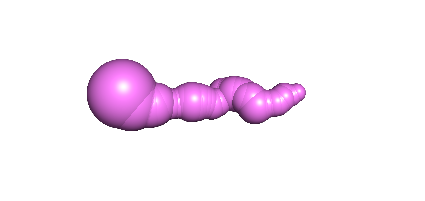


**SI-Figure 6** Visual representation of the tunnels oriented all in the same way. The left side of the tunnels is the entrance of the tunnel, and the right side is the end of the tunnel where the S2 domain starts. Colour coding used is the same as before.


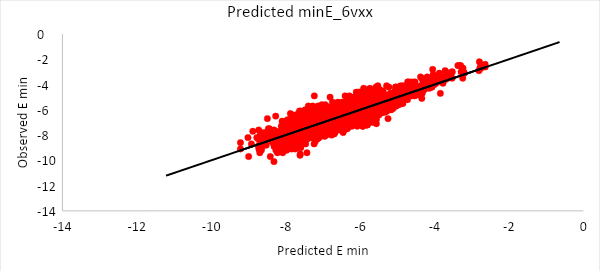

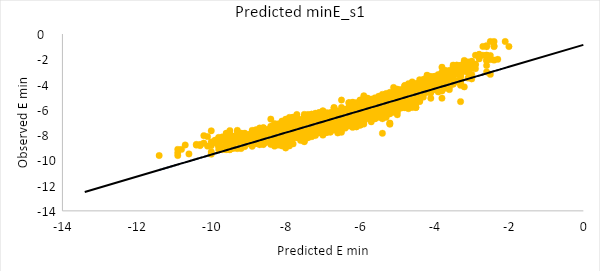

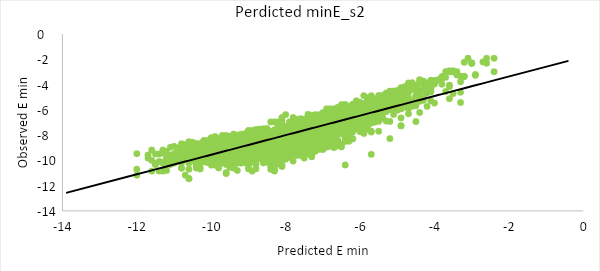

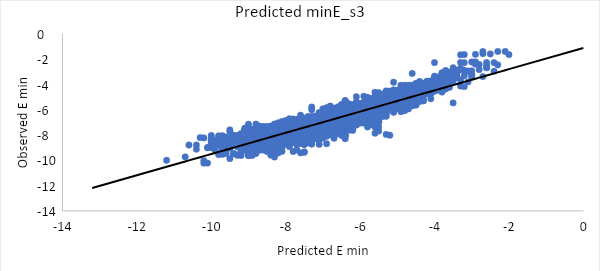

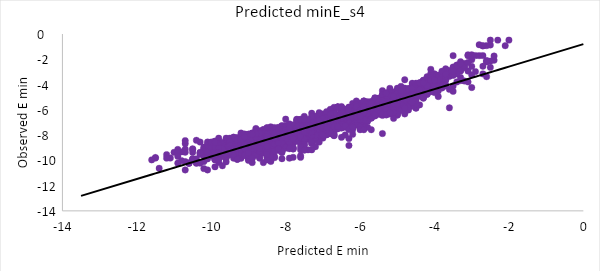

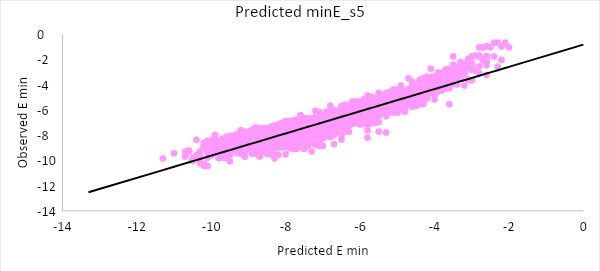


**SI-Figure 7** The plot of predicted *versus* observed minimal binding energies (E min) obtained for all the states. Here we show that the least correlating protein is the cryo-EM structure 6VXX with R^2^ = 0.77 (top) and the best correlating protein conformation representative of cluster s4 with R^2^ = 0.89 (bottom). Compounds showing the strongest binding to the particular state of the spike protein are located at the left-down part of the plot.


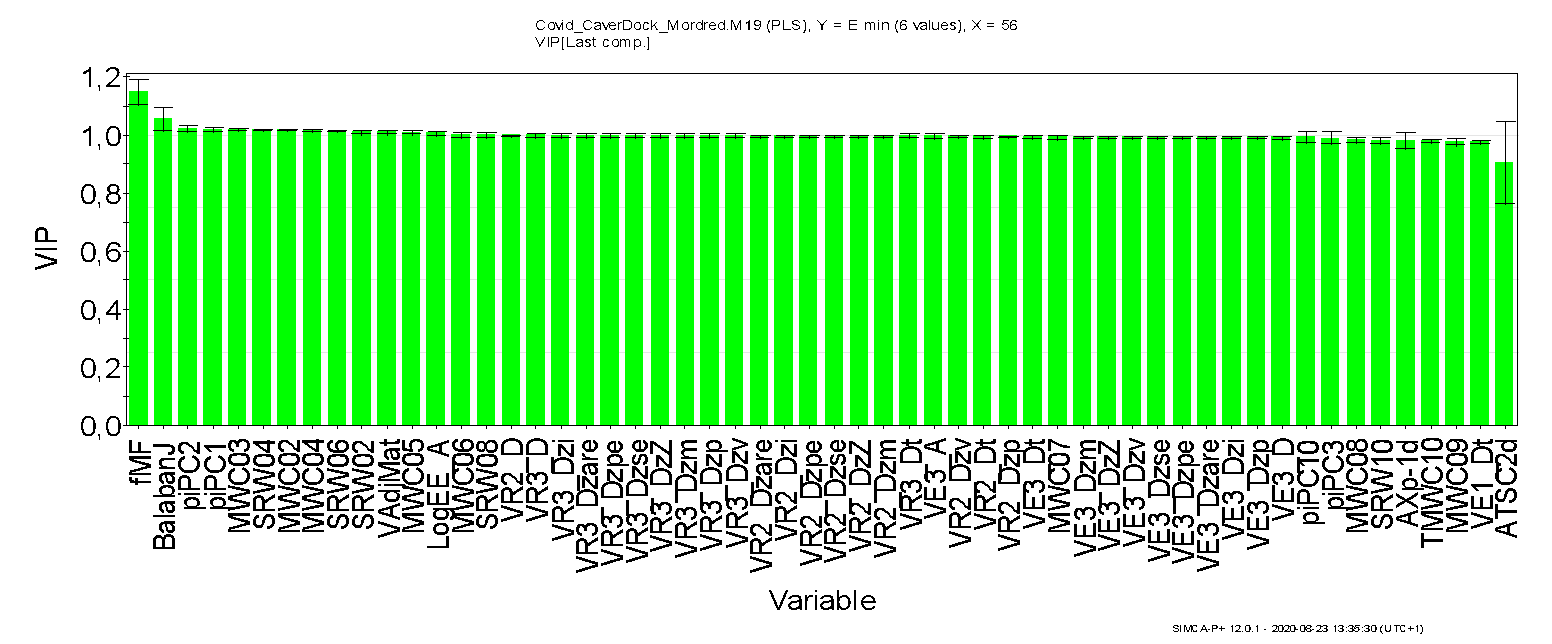


**SI-Figure 8** Variable importance on the projection plot. VIP values quantify the contribution of individual variables to the explanation of minimal binding energies simultaneously for all six protein states. The larger value means the higher impact of the variable on the experimental observable.

**SI-Table 1** Top ten ranked drugs obtained from the PCA and PLS analyses. Drugs are represented with their ZINC codes. Structures are represented through their PDB ID and the number of the cluster as before. Percentage of time in contact with 1, 2 or 3 monomers (C1, C2 and C3, respectively) are presented per structure for every drug.

|  | **6VXX** | | | **State S1** | | | **State S2** | | | **State S3** | | | **State S4** | | | **State S5** | | |
| --- | --- | --- | --- | --- | --- | --- | --- | --- | --- | --- | --- | --- | --- | --- | --- | --- | --- | --- |
|  | C1 | C2 | C3 | C1 | C2 | C3 | C1 | C2 | C3 | C1 | C2 | C3 | C1 | C2 | C3 | C1 | C2 | C3 |
| **ZINC000169289767** | 0.0 | 9.5 | 90.5 | 5.3 | 19.7 | 75.0 | 0.0 | 3.4 | 96.6 | 0.7 | 24.5 | 74.8 | 0.2 | 1.6 | 98.2 | 2.9 | 19.7 | 77.5 |
| **ZINC000164760756** | 0.0 | 13.4 | 86.6 | 7.0 | 19.4 | 73.6 | 0.2 | 11.5 | 88.3 | 3.8 | 18.6 | 77.6 | 0.5 | 5.1 | 94.5 | 3.2 | 21.1 | 75.7 |
| **ZINC000936069565** | 0.0 | 19.5 | 80.5 | 5.1 | 24.7 | 70.2 | 0.0 | 9.9 | 90.1 | 2.6 | 26.4 | 71.0 | 1.2 | 3.9 | 94.9 | 6.1 | 15.0 | 78.9 |
| **ZINC000028639340** | 0.0 | 7.1 | 92.9 | 3.7 | 19.4 | 77.0 | 0.0 | 12.1 | 87.9 | 0.5 | 21.4 | 78.1 | 0.0 | 12.2 | 87.8 | 6.1 | 20.5 | 73.4 |
| **ZINC000027990463** | 0.4 | 20.9 | 78.8 | 4.5 | 21.4 | 74.2 | 0.0 | 9.3 | 90.7 | 1.7 | 19.3 | 79.1 | 0.0 | 2.3 | 97.7 | 3.5 | 23.4 | 73.1 |
| **ZINC000096015174** | 0.4 | 14.1 | 85.5 | 3.7 | 25.8 | 70.5 | 0.0 | 16.7 | 83.3 | 7.4 | 17.9 | 74.8 | 0.0 | 5.1 | 94.9 | 3.8 | 11.9 | 84.4 |
| **ZINC000068204830** | 0.0 | 16.3 | 83.8 | 3.7 | 16.6 | 79.8 | 0.0 | 5.5 | 94.5 | 1.9 | 21.2 | 76.9 | 0.0 | 5.3 | 94.7 | 3.5 | 20.5 | 76.0 |
| **ZINC000003934128** | 0.0 | 15.6 | 84.4 | 8.4 | 22.5 | 69.1 | 0.0 | 9.1 | 90.9 | 1.4 | 21.0 | 77.6 | 0.0 | 6.7 | 93.3 | 6.9 | 20.2 | 72.8 |
| **ZINC000003978005** | 0.7 | 21.6 | 77.7 | 9.0 | 25.6 | 65.5 | 0.4 | 16.8 | 82.7 | 2.9 | 20.5 | 76.7 | 1.2 | 9.0 | 89.9 | 7.8 | 35.8 | 56.4 |
| **ZINC000003995616** | 0.4 | 22.3 | 77.4 | 8.4 | 25.6 | 66.0 | 0.0 | 17.3 | 82.7 | 3.1 | 20.7 | 76.2 | 1.2 | 5.5 | 93.3 | 9.0 | 35.3 | 55.8 |

**SI-Table 2** Generic drug names and corresponding ZINC codes for the top-ranked drugs.

| **Generic name** | **Zinc Code** |
| --- | --- |
| Trypan blue | [ZINC000169289767](https://www.drugbank.ca/drugs/DB09158) |
| Simeprevir | [ZINC000164760756](https://www.drugbank.ca/drugs/DB06290) |
| Glecaprevir | [ZINC000936069565](https://www.drugbank.ca/drugs/DB13879) |
| Posaconazole | [ZINC000028639340](https://www.drugbank.ca/drugs/DB01263) |
| Lomitapide | [ZINC000027990463](https://www.drugbank.ca/drugs/DB08827) |
| Glycyrrhizinate Dipotassium | [ZINC000096015174](https://www.drugbank.ca/drugs/DB13751) |
| Daclatasvir | [ZINC000068204830](https://www.drugbank.ca/drugs/DB09102) |
| Temoporfin | [ZINC000003934128](https://www.drugbank.ca/drugs/DB11630) |
| Dihydroergotamine | [ZINC000003978005](https://www.drugbank.ca/drugs/DB00320) |
| Dihydroergocristine | [ZINC000003995616](https://www.drugbank.ca/drugs/DB13345) |

**SI-Table 3** Free energy of binding (ΔG_bind_^Total^) obtained from the MM/GBSA method for the top-ranked drugs with the spike glycoprotein using the minimum-energy complexes with the 6VXX structure.

| **Drug name** | **ZINC code** | **CaverDock**  **ΔG_bind_**  **(kcal/mol)** | **MM/GBSA**  **ΔG_bind_^Total^**  **(kcal/mol)** |
| --- | --- | --- | --- |
| Daclatasvir | ZINC000068204830 | -7.8 | -46.1 |
| Dihydroergocristine | ZINC000003995616 | -9.2 | -42.1 |
| Lomitapide | ZINC000027990463 | -10.1 | -38.2 |
| Posaconazole | ZINC000028639340 | -9.2 | -37.6 |
| Dihydroergotamine | ZINC000003978005 | -9.1 | -36.2 |
| Glecaprevir | ZINC000936069565 | -9.1 | -35.5 |
| Temoporfin | ZINC000003934128 | -8.3 | -35.2 |
| Glycyrrhizinate Dipotassium | ZINC000096015174 | -9.0 | -30.1 |
| Simeprevir | ZINC000164760756 | -8.7 | -27.3 |
| Trypan blue | ZINC000169289767 | -9.7 | 45.4 |

**SI-Table 4** Free energy of binding obtained from the MM/GBSA method for the top-ranked drugs with the spike glycoprotein using the minimum-energy complexes with the 6VXX structure^a^

| **Drug/**  **Residues** | **ΔG_bind_** | **Drug/**  **Residues** | **ΔG_bind_** | **Drug/**  **Residues** | **ΔG_bind_** | **Drug/**  **Residues** | **ΔG_bind_** | **Drug/**  **Residues** | **ΔG_bind_** |
| --- | --- | --- | --- | --- | --- | --- | --- | --- | --- |
| **Daclatasvir** | | **Dihydroergocristine** | | **Lomitapide** | | **Posaconazole** | | **Dihydroergotamine** | |
| **ZINC000068204830** | | **ZINC000003995616** | | **ZINC000027990463** | | **ZINC000028639340** | | **ZINC000003978005** | |
| ***Total*** | ***-46.1*** | ***Total*** | ***-42.1*** | ***Total*** | ***-38.2*** | ***Total*** | ***-37.6*** | ***Total*** | ***-36.2*** |
| ARG A 995 | -2.03 | TYR A 756 | -1.39 | PHE A 970 | -1.71 | TYR A 756 | -3.34 | PHE A 970 | -2.23 |
| THR A 998 | -2.84 | PHE A 970 | -1.27 | ARG A 995 | -1.17 | PHE A 759 | -0.55 | GLY A 971 | -1.10 |
| GLN A1002 | -3.94 | GLY A 971 | -0.84 | THR A 998 | -5.81 | GLY A 971 | -1.35 | VAL A 991 | -1.06 |
| GLN A1005 | -2.72 | GLU A 990 | -0.59 | GLY A 999 | -1.45 | VAL A 991 | -0.51 | ASP A 994 | -1.44 |
| THR A1006 | -1.73 | VAL A 991 | -5.21 | GLN A1002 | -5.15 | ASP A 994 | -2.20 | ARG A 995 | -9.55 |
| THR A1009 | -1.43 | ASP A 994 | -1.63 | GLN A1005 | -5.52 | ARG A 995 | -5.19 | THR A 998 | -3.63 |
| TYR B 756 | -1.96 | ARG A 995 | -5.90 | THR A1006 | -1.28 | THR A 998 | -3.93 | GLY A 999 | -0.61 |
| PHE B 970 | -0.55 | THR A 998 | -6.27 | VAL A1008 | -0.62 | PRO B 986 | -0.90 | GLN A1002 | -1.00 |
| ASP B 994 | -1.39 | TYR B 756 | -1.73 | THR A1009 | -1.62 | PRO B 987 | -3.14 | LEU B 752 | -1.15 |
| ARG B 995 | -3.97 | PHE B 970 | -0.65 | TYR B 756 | -1.55 | GLU B 990 | -1.73 | TYR B 756 | -1.89 |
| THR B 998 | -6.10 | VAL B 991 | -0.94 | PHE B 759 | -0.63 | VAL B 991 | -3.58 | GLU B 990 | -2.97 |
| LEU B1001 | -0.65 | ASP B 994 | -2.52 | ARG B 995 | -1.08 | ASP B 994 | -1.99 | VAL B 991 | -5.10 |
| GLN B1002 | -5.14 | ARG B 995 | -2.67 | THR B 998 | -4.92 | ARG B 995 | -1.43 | ASP B 994 | -10.59 |
| GLN B1005 | -4.49 | THR B 998 | -3.76 | GLY B 999 | -0.56 | THR B 998 | -1.56 | ARG B 995 | -1.81 |
| THR B1006 | -3.62 | TYR C 756 | -0.59 | GLN B1002 | -5.08 | PRO C 412 | -0.52 | THR B 998 | -2.21 |
| THR B1009 | -2.53 | PHE C 970 | -1.64 | GLN B1005 | -6.68 | GLY C 413 | -0.82 | ASP C 427 | -1.97 |
| GLN B1010 | -0.94 | ARG C 995 | -7.20 | THR B1006 | -2.80 | ASP C 427 | -3.49 | ASP C 428 | -0.55 |
| TYR C 756 | -0.70 | THR C 998 | -7.65 | THR B1009 | -0.92 | ASP C 428 | -0.89 | ARG C 995 | -0.76 |
| LEU C 763 | -0.53 | GLY C 999 | -0.66 | GLN B1010 | -0.51 | PHE C 970 | -1.56 |  |  |
| VAL C 991 | -1.65 | GLN C1002 | -0.65 | THR C 998 | -3.32 | ASP C 994 | -1.01 |  |  |
| ASP C 994 | -2.41 |  |  | GLN C1002 | -5.18 | ARG C 995 | -3.84 |  |  |
| ARG C 995 | -1.25 |  |  | GLN C1005 | -4.33 | THR C 998 | -5.21 |  |  |
| THR C 998 | -1.53 |  |  | THR C1006 | -2.17 | GLY C 999 | -1.04 |  |  |
| GLN C1002 | -3.56 |  |  | THR C1009 | -2.93 | GLN C1002 | -1.04 |  |  |
| GLN C1005 | -4.91 |  |  | GLN C1010 | -2.05 |  |  |  |  |
| THR C1006 | -2.23 |  |  |  |  |  |  |  |  |
| THR C1009 | -2.93 |  |  |  |  |  |  |  |  |
|  |  |  |  |  |  |  |  |  |  |

**SI-Table 4** *(cont.)*

| **Drug/**  **Residues** | **ΔG_bind_** | **Drug/**  **Residues** | **ΔG_bind_** | **Drug/**  **Residues** | **ΔG_bind_** | **Drug/**  **Residues** | **ΔG_bind_** | **Drug/**  **Residues** | **ΔG_bind_** |
| --- | --- | --- | --- | --- | --- | --- | --- | --- | --- |
| Glecaprevir | | Temoporfin | | Glycyrrhizinate Dipotassium | | Simeprevir | | Trypan blue | |
| ZINC000936069565 | | ZINC000003934128 | | ZINC000096015174 | | ZINC000164760756 | | ZINC000169289767 | |
| ***Total*** | ***-35.5*** | ***Total*** | ***-35.2*** | ***Total*** | ***-30.1*** | ***Total*** | ***-27.3*** | ***Total*** | ***45.4*** |
| ASP A 994 | 0.65 | LEU A 752 | -0.69 | TYR A 756 | -1.36 | ASP A 428 | -0.67 | TYR A 756 | -4.88 |
| ARG A 995 | -2.20 | TYR A 756 | -2.19 | VAL A 991 | -3.76 | VAL A 991 | -1.41 | PHE A 970 | -1.07 |
| PHE B 970 | -2.06 | GLU A 990 | -1.99 | ASP A 994 | -1.17 | ASP A 994 | -1.45 | ASP A 994 | -3.49 |
| GLY B 971 | -0.88 | VAL A 991 | -4.64 | ARG A 995 | -4.11 | ARG A 995 | -3.05 | ARG A 995 | -3.07 |
| VAL B 991 | -3.91 | ASP A 994 | -8.86 | THR A 998 | -7.78 | THR A 998 | -1.16 | THR A 998 | -2.74 |
| ASP B 994 | -2.07 | ARG A 995 | -3.02 | VAL B 991 | -1.84 | GLY B 971 | -1.73 | TYR B 756 | -1.54 |
| ARG B 995 | -8.03 | THR A 998 | -5.04 | ARG B 995 | -4.21 | ALA B 972 | -0.99 | PHE B 970 | -1.86 |
| LEU B 996 | -0.51 | VAL B 991 | -3.17 | THR B 998 | -1.02 | ILE B 973 | -1.65 | GLY B 971 | -1.71 |
| THR B 998 | -1.56 | GLN B 992 | -0.52 | PHE C 970 | -1.28 | VAL B 991 | -0.63 | ASP B 994 | -1.92 |
| GLY B 999 | -1.01 | ASP B 994 | -3.09 | VAL C 991 | -5.33 | ASP B 994 | -1.28 | ARG B 995 | -8.03 |
| GLN 1002 | -1.40 | ARG B 995 | -3.02 | GLN C 992 | -0.53 | ARG B 995 | -5.92 | THR B 998 | -3.46 |
| TYR C 756 | -2.17 | PHE C 970 | -2.60 | ASP C 994 | -2.71 | THR B 998 | -1.10 | TYR C 756 | -0.86 |
| PRO C 987 | -0.74 | GLY C 971 | -1.87 | ARG C 995 | -7.34 | GLN C 755 | -1.11 | PHE C 970 | -0.68 |
| GLU C 990 | -1.10 | ARG C 995 | -6.11 | THR C 998 | -3.78 | GLU C 990 | -1.19 | GLY C 971 | -0.88 |
| VAL C 991 | -3.94 | THR C 998 | -2.07 |  |  | VAL C 991 | -5.56 | VAL C 991 | -3.72 |
| ASP C 994 | -2.84 |  |  |  |  | ASP C 994 | -2.94 | ASP C 994 | -2.41 |
| ARG C 995 | -4.61 |  |  |  |  | ARG C 995 | -10.55 | ARG C 995 | -3.85 |
| THR C 998 | -8.01 |  |  |  |  | THR C 998 | -1.80 | THR C 998 | -2.03 |
|  |  |  |  |  |  |  |  |  |  |

^a^The total binging energy (**Δ**G_bind_^Total^) is displayed in bold and italic and the **Δ**G_bind_ contributions by individual residues are listed below; only interactions <-0.5 kcal/mol are presented.

| **SI-Table 5** Top ten drugs combinations of the Tanimoto similarity, the numbers of atoms and bonds in the most common substructure and the SMARTS code of those structures. | | | | | |
| --- | --- | --- | --- | --- | --- |
| **Molecule 1** | **Molecule 2** | **Tanimoto similarity**  **score** | **Number of atoms in  most common substructure** | **Number of bonds in  most common substructure** | **SMARTS code** |
| Dihydroergotamine | Dihydroergocristine | 0.98 | 43 | 50 | [#6]-!@[#7]-@1-@[#6]-@[#6](-@[#6]-@[#6]-@2-@[#6]:3:[#6]:[#6]:[#6]:[#6]:4:[#7]:[#6]:[#6](-@[#6]-@[#6]-@1-@2):[#6]:3:4)-!@[#6](-!@[#7]-!@[#6]-@1(-@[#8]-@[#6]-@2(-@[#6]-@3-@[#6]-@[#6]-@[#6]-@[#7]-@3-@[#6](-@[#6](-@[#7]-@2-@[#6]-@1=!@[#8])-!@[#6]-!@[#6]:1:[#6]:[#6]:[#6]:[#6]:[#6]:1)=!@[#8])-!@[#8])-!@[#6])=!@[#8] |
| Glecaprevir | Dihydroergotamine | 0.70 | 15 | 15 | [#6](-@[#6])-@[#8]-@[#6]-@[#7]-@[#6](-!@[#6]-!@[#6])-@[#6](=!@[#8])-@[#7]-@1-@[#6]-@[#6]-@[#6]-@[#6]-@1 |
| Glecaprevir | Dihydroergocristine | 0.71 | 15 | 15 | [#6](-@[#7]-@1-@[#6]-@[#6]-@[#6]-@[#6]-@1)(-@[#6](-@[#7]-@[#6]-@[#8]-@[#6]-@[#6])-!@[#6]-!@[#6])=!@[#8] |
| Lomitapide | Daclatasvir | 0.39 | 13 | 14 | [#6]:1:[#6]:[#6]:[#6]:[#6]:[#6]:1-!@[#6]:1:[#6]:[#6]:[#6](:[#6]:[#6]:1)-!@[#6] |
| Lomitapide | Temoporfin | 0.35 | 13 | 13 | [#6]-!@[#6]:[#6]:[#6]:[#6]:[#6]:[#6]-!@[#6]:1:[#6]:[#6]:[#6]:[#6]:[#6]:1 |
| Trypan blue | Lomitapide | 0.37 | 12 | 13 | [#6]:1:[#6]:[#6]:[#6]:[#6]:[#6]:1-!@[#6]:1:[#6]:[#6]:[#6]:[#6]:[#6]:1 |
| Trypan blue | Daclatasvir | 0.31 | 12 | 13 | [#6]:1:[#6]:[#6]:[#6](-!@[#6]:2:[#6]:[#6]:[#6]:[#6]:[#6]:2):[#6]:[#6]:1 |
| Trypan blue | Temoporfin | 0.31 | 12 | 12 | [#6]:1:[#6]:[#6]:[#6]:[#6](:[#6]:1)-!@[#6](:[#6]:[#6]:[#6]:[#6]):[#6] |
| Simeprevir | Glecaprevir | 0.69 | 12 | 13 | [#6]-@1(-!@[#6](-!@[#7]-!@[#16](-!@[#6]-@2-@[#6]-@[#6]-@2)(=!@[#8])=!@[#8])=!@[#8])-@[#6]-@[#6]-@1 |
| Simeprevir | Temoporfin | 0.38 | 12 | 11 | [#6](:[#6](-!@[#6]):[#6](:[#6]:[#6]:[#6]:[#6]-!@[#6]):[#7]):[#6]:[#6] |
| Glecaprevir | Temoporfin | 0.37 | 12 | 11 | [#6](:[#6]:[#6]:[#6]:[#6]:[#7]):[#6]:[#7]:[#6](-@[#6]-@[#6]):[#6] |
| Daclatasvir | Temoporfin | 0.33 | 12 | 12 | [#6]:1:[#6]:[#6]:[#6](-!@[#6](:[#6]:[#6]:[#6]):[#6]:[#6]):[#6]:[#6]:1 |
| Temoporfin | Dihydroergotamine | 0.37 | 12 | 11 | [#6](-@[#6]-@[#6]-@[#6]):[#6]:[#6]:[#6]:[#6]:[#6]:[#6]:[#6]:[#7] |
| Temoporfin | Dihydroergocristine | 0.38 | 12 | 11 | [#6]-@[#6]-@[#6]-@[#6]:[#6]:[#6]:[#6]:[#6]:[#6]:[#6]:[#6]:[#7] |
| Simeprevir | Glycyrrhizinate | 0.40 | 11 | 10 | [#6](-@[#6]-@[#6]-@[#6]=@[#6]-@[#6]-@[#6]-@[#6]-@[#6])-!@[#6]=!@[#8] |
| Trypan blue | Simeprevir | 0.41 | 10 | 10 | [#6]:1:[#6]:[#6]:[#6](:[#6]:[#6]:1):[#6](:[#6]:[#6])-!@[#8] |
| Simeprevir | Daclatasvir | 0.44 | 10 | 9 | [#6]:[#6]:[#6]:[#6]:[#6]:[#6]-!@[#6]:[#7]:[#6]-!@[#6] |
| Simeprevir | Dihydroergotamine | 0.66 | 10 | 9 | [#6]-!@[#7](-@[#6]-@[#6]-@[#6])-@[#6]-@[#6]-@[#6]-@[#6]-@[#6] |
| Simeprevir | Dihydroergocristine | 0.68 | 10 | 9 | [#6](-@[#6]-@[#7](-!@[#6])-@[#6]-@[#6]-@[#6])-@[#6]-@[#6]-@[#6] |
| Glycyrrhizinate | Dihydroergotamine | 0.39 | 10 | 9 | [#6]-@[#6](-@[#6]-@[#6](-!@[#6]=!@[#8])-@[#6])-@[#6]-@[#6]-@[#6] |
| Glycyrrhizinate | Dihydroergocristine | 0.40 | 10 | 9 | [#6](-!@[#6](-@[#6])-@[#6]-@[#6](-@[#6]-@[#6]-@[#6])-@[#6])=!@[#8] |
| Trypan blue | Posaconazole | 0.40 | 8 | 7 | [#7]-!@[#6](:[#6]:[#6]):[#6]:[#6]:[#6]-!@[#7] |
| Trypan blue | Dihydroergotamine | 0.42 | 8 | 8 | [#6]:1:[#6]:[#6]:[#6]:[#6]:[#6]:1:[#6]:[#6] |
| Trypan blue | Dihydroergocristine | 0.42 | 8 | 8 | [#6]:1:[#6]:[#6]:[#6]:[#6]:[#6]:1:[#6]:[#6] |
| Simeprevir | Posaconazole | 0.58 | 8 | 8 | [#6]:1:[#6](-!@[#8]-!@[#6]):[#6]:[#6]:[#6]:[#6]:1 |
| Simeprevir | Lomitapide | 0.48 | 8 | 7 | [#6]-!@[#6]:[#6]:[#6]:[#6]:[#6]:[#6]-!@[#6] |
| Posaconazole | Dihydroergotamine | 0.62 | 8 | 8 | [#6]-!@[#6]-!@[#6]:1:[#6]:[#6]:[#6]:[#6]:[#6]:1 |
| Posaconazole | Dihydroergocristine | 0.63 | 8 | 8 | [#6]-!@[#6]-!@[#6]:1:[#6]:[#6]:[#6]:[#6]:[#6]:1 |
| Lomitapide | Dihydroergotamine | 0.54 | 8 | 8 | [#6]:1(-@[#6]-@[#6]):[#6]:[#6]:[#6]:[#6]:[#6]:1 |
| Lomitapide | Dihydroergocristine | 0.54 | 8 | 8 | [#6]-@[#6]-@[#6]:1:[#6]:[#6]:[#6]:[#6]:[#6]:1 |
| Glecaprevir | Lomitapide | 0.49 | 7 | 6 | [#8]=!@[#6](-!@[#6])-!@[#7]-!@[#6](-@[#6])-@[#6] |
| Glecaprevir | Daclatasvir | 0.45 | 7 | 6 | [#6](-!@[#7]-!@[#6]-!@[#6](-!@[#7])=!@[#8])=!@[#8] |
| Posaconazole | Lomitapide | 0.48 | 7 | 7 | [#6]-!@[#6]:1:[#6]:[#6]:[#6]:[#6]:[#6]:1 |
| Posaconazole | Glycyrrhizinate acid | 0.36 | 7 | 6 | [#6]-!@[#6](-!@[#6])-@[#6]-@[#6](-@[#6])-!@[#6] |
| Posaconazole | Daclatasvir | 0.39 | 7 | 7 | [#6]:1(:[#6]:[#6]:[#6]:[#6]:[#6]:1)-!@[#6] |
| Posaconazole | Temoporfin | 0.35 | 7 | 7 | [#6]:1:[#6](-!@[#8]):[#6]:[#6]:[#6]:[#6]:1 |
| Daclatasvir | Dihydroergotamine | 0.46 | 7 | 7 | [#6]-!@[#6]:1:[#6]:[#6]:[#6]:[#6]:[#6]:1 |
| Daclatasvir | Dihydroergocristine | 0.47 | 7 | 7 | [#6]-!@[#6]:1:[#6]:[#6]:[#6]:[#6]:[#6]:1 |
| Trypan blue | Glecaprevir | 0.39 | 6 | 6 | [#6]:1:[#6]:[#6]:[#6]:[#6]:[#6]:1 |
| Glecaprevir | Posaconazole | 0.58 | 6 | 6 | [#6]:1:[#6]:[#6]:[#6]:[#6]:[#6]:1 |
| Glecaprevir | Glycyrrhizinate acid | 0.39 | 6 | 5 | [#6](-@[#6])-@[#6]-@[#6]-!@[#6]=!@[#8] |
| Lomitapide | Glycyrrhizinate acid | 0.32 | 6 | 5 | [#6]-!@[#6](-!@[#6]=!@[#8])(-@[#6])-@[#6] |
| Glycyrrhizinate | Daclatasvir | 0.30 | 5 | 4 | [#6]-@[#6]-@[#6]-@[#6]-!@[#6] |
| Glycyrrhizinate | Temoporfin | 0.25 | 4 | 3 | [#6]-@[#6]-@[#6]-@[#6] |
| Trypan blue | Glycyrrhizinate acid | 0.26 | 2 | 1 | [#6]-!@[#6] |
